# Prioritizing Pharmaceuticals for Environmental Monitoring in Greece: A Comprehensive Review of Consumption, Occurrence, and Ecological Risk

**DOI:** 10.3390/toxics14010045

**Published:** 2025-12-30

**Authors:** Konstantina-Roxani Chatzipanagiotou, Adamantia Bon, Foteini Petrakli, George Antonaropoulos, Elias P. Koumoulos

**Affiliations:** IRES—Innovation in Research and Engineering Solutions SNC, Silversquare Europe, Square de Meeûs 35, 1000 Brussels, Belgium; kchatzip@innovation-res.eu (K.-R.C.); adamantiabon@innovation-res.eu (A.B.); gantonaropoulos@innovation-res.eu (G.A.)

**Keywords:** environmental monitoring, pharmaceuticals, ecological risk assessment, emerging contaminants, Greece, wastewater

## Abstract

Pharmaceuticals are increasingly recognized as contaminants of emerging concern, yet monitoring strategies often do not reflect actual consumption patterns or ecological risk. Greece presents a particularly relevant case due to high pharmaceutical use and fragmented monitoring data. In the present study, 359 pharmaceuticals, metabolites, and transformation products were reviewed, as reported in monitoring studies in Greek wastewater, surface waters, and drinking water. Consumption data (from the Organization for Economic Co-operation and Development, OECD), environmental occurrence (from 55 studies), and ecotoxicity thresholds (i.e., from the NORMAN Database) were integrated to calculate risk quotients (RQs) and assess monitoring gaps. RQ values were derived for 241 compounds: 38 (16%) high-risk, 60 (25%) medium-risk, and 143 (59%) low-risk. High-risk substances included several NSAIDs, macrolide and fluoroquinolone antibiotics, synthetic hormones, contrast agents, and triclosan. Major under-monitoring was observed for widely consumed classes A and B, while antibiotics, NSAIDs, antidepressants, and analgesics were disproportionately targeted. Several metabolites showed higher RQs than their parent compounds but were rarely analyzed. These findings reveal significant mismatches between pharmaceutical use, environmental occurrence, and ecological risk in Greece. Results support adopting risk-based prioritization for environmental monitoring and align with ongoing updates to EU water policy.

## 1. Introduction

The global pharmaceutical market has undergone a significant increase over the past two decades. According to worldwide revenue data, the global pharmaceutical market was valued at approximately USD 1.42 trillion at the end of 2021, while in 2001, the market was valued at USD 390 billion [1]. This trend is also evident based on Research and Development (R&D) efforts within the pharmaceutical industry. For example, the total global spending on pharmaceutical R&D has increased from USD 137 billion in 2012 to USD 238 billion in 2021 [2], although such efforts are not reflected in the number of novel drug products approved annually, which tends to fluctuate between years and does not follow a similar increasing trend over time [3]. More recent data on global R&D spending for large pharmaceutical companies have been reported, with USD 190 billion in 2024, which increased by over 16% compared to the previous year [4]. With the projected increase in the human population [5], it is expected that the pharmaceutical sector and discovery of new drugs will continue to grow in the coming decades.

**Figure 1:**
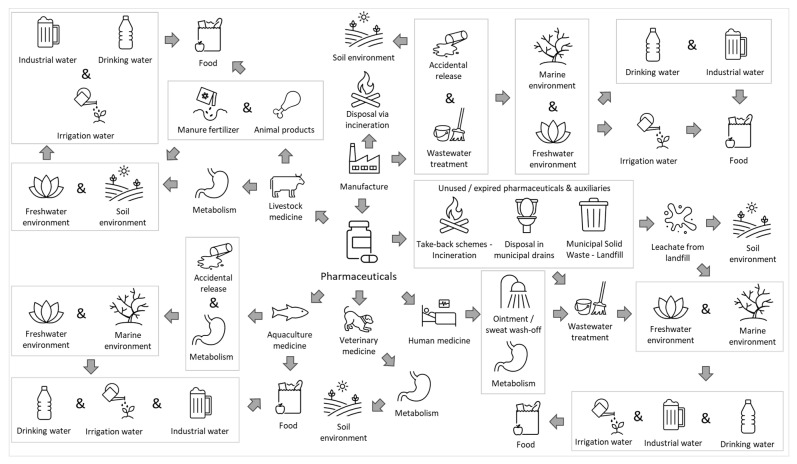
Routes of exposure to pharmaceuticals and environmental release along their life cycle.

While the improved availability of pharmaceutical products is undoubtedly linked to an improved quality of life, several researchers have highlighted the negative aspects of pharmaceutical production in terms of environmental degradation [6,7,8,9,10]. In an attempt to make the pharmaceutical sector “greener”, significant efforts have been made to improve the production process, such as by replacing solvents [11], switching catalysts (e.g., from chemical catalysis to enzymatic catalysis) [12], decreasing energy consumption [13], and switching from batch to continuous manufacturing [14]. However, the environmental impact of pharmaceuticals does not stop at production. One important negative effect of increased production and consumption of pharmaceuticals is the release of active pharmaceutical ingredients (APIs), metabolites, and degradation products (e.g., via wastewater treatment) to the environment [15,16,17,18,19,20,21]. Several routes have been described for how pharmaceuticals end up in the environment (see Figure 1). Among these, it has been estimated that a large percentage originates from the consumption of pharmaceuticals, which can be partially metabolized by the human body, and the rest is eliminated via feces and urine [22].

Once released, pharmaceuticals and their metabolites enter wastewater treatment plants (WWTPs), where, depending on the technologies used in each facility, the chemical structure of each individual compound, as well as external factors such as climate, they are (bio)degraded, adsorbed on solids/sludge, transformed, and finally released to the environment via the WWTP effluent [23,24,25,26,27,28,29,30]. A concentrated stream of (partially metabolized) pharmaceutical waste includes hospitals, nursing homes, and hospices [31,32,33,34,35,36,37,38,39,40,41], facilities that may include a separate wastewater treatment unit [41,42,43,44]. Eventually, the (treated) wastewater from these sources, as well as individual households, is led to municipal wastewater treatment plants [31,32,33,34,35,36,37,38,39,40,41], where it undergoes most commonly primary and secondary wastewater treatment, while some WWTPs may also include more advanced tertiary treatment options, such as chlorination, ozonation, or adsorption with activated carbon [45,46,47]. In addition to pharmaceuticals that have been consumed and excreted, another important source of pharmaceuticals in WWTPs includes improperly disposed of (expired) products, which may be directly discarded in the sink or toilet [48].

Many scientific studies have been conducted on the fate and risk of pharmaceutical products in the environment [16,17,49,50,51,52,53,54,55,56]. While pharmaceuticals are detected in the environment at low concentrations, up to a few μg/L, they are considered emerging pollutants of high importance [57], given the fact that they are designed to result in significant effects on organisms, even at low concentrations [21]. Another factor that makes pharmaceuticals an important threat to natural ecosystems is their tendency to bioaccumulate, thus resulting in increasing concentrations in the biomass along trophic chains [58,59,60]. In addition to the negative effects of released pharmaceuticals across the food web, the consumption of food exposed to pharmaceuticals can also pose health risks to humans [61,62]. Nevertheless, despite the known risks of pharmaceuticals ending up in the environment, harmonized legislation on a European and global scale has not included, as of yet, specific targets for the maximum allowable concentration of pharmaceuticals in water, and, with a few exceptions, there is no recommendation for the monitoring of wastewater effluents or natural waters in terms of pharmaceuticals. According to the most recent update in 2025, the EU Surface Water Watch List under the Water Framework Directive (2000/60/EC) was revised to include several new pharmaceutical substances, namely fluoxetine, propranolol, and a group of tetracycline antibiotics (tetracycline and oxytetracycline), alongside three pharmaceutical azole antifungal agents (climbazole, ketoconazole, and itraconazole). Previously listed substances such as sulfamethoxazole, trimethoprim, venlafaxine and its metabolite O-desmethylvenlafaxine, three pharmaceutical azoles (clotrimazole, fluconazole, miconazole), and the fungicides dimoxystrobin and famoxadone were removed following the completion of the monitoring period and upcoming risk assessments. On the other hand, the fungicide azoxystrobin added in 2022 was removed due to the high-quality monitoring data available, while the veterinary pharmaceutical fipronil, the antibiotics clindamycin and ofloxacin, and the human pharmaceutical metformin and its metabolite guanylurea remained on the list for further monitoring [63]. Prioritization of pharmaceutical substances was also recently proposed for the Urban Wastewater Treatment Directive (91/271/EEC), which, according to the most recently published Directive (EU) 2024/3019, will require monitoring of amisulpride, carbamazepine, citalopram, clarithromycin, diclofenac, hydrochlorothiazide, metoprolol, venlafaxine, candesartan, irbesartan, and benzotriazole and its derivatives (which are commonly used as drug precursors) [57].

In order to propose a list of priority pharmaceutical substances for monitoring in environmental waters and treatment in WWTPs, the following criteria should be considered:

(a) The total amount of sales/consumption for specific pharmaceuticals in a certain region should be known to predict the compounds with the highest expected environmental concentrations. Interestingly, it has been reported that pharmaceutical removal efforts are often not motivated by market trends in pharmaceutical consumption [64].
(b) The metabolism of pharmaceuticals needs to be considered in order to determine whether unmetabolized pharmaceuticals or their metabolites are expected to be found in wastewater and natural waters receiving the final effluent of WWTPs.
(c) Once excreted by the patient, the unmetabolized compounds and metabolites can undergo transformation in the WWTP, which can result in complete elimination from the aqueous phase (i.e., via degradation or adsorption), or the formation of transformation products, particularly when advanced water treatment techniques are applied (e.g., chlorination, ozonation) [23,24,25,26,27,28,29,30]. To that end, valuable information can be gained from scientific literature and databases concerning the detection of pharmaceuticals, metabolites, and transformation products in the environment and in WWTPs.
(d) Finally, the ecotoxicity and bioaccumulation potential of these compounds on various organisms ought to be considered to prioritize pharmaceuticals with the most severe expected effects in ecosystems and human health.

Although pharmaceuticals as emerging contaminants constitute a global problem, an assessment taking into account these criteria cannot be realistically applied on a global or European level, due to the immense number of publications related to the presence of pharmaceuticals in natural waters, groundwater, drinking water, and wastewater. Another limiting factor in performing such an evaluation on the global level is the difference in pharmaceutical consumption patterns [65] and disposal practices [66] encountered in different countries. To that end, Greece is selected as a case study for several reasons. Firstly, considering the high levels of pharmaceutical consumption among European countries, it is reasonable to assume increased pressure on natural waters and ecosystems in Greece from released pharmaceutical compounds, metabolites, and transformation products. A recent statistical analysis published by the Organization for Economic Co-operation and Development (OECD) revealed that, among the 41 countries considered (European and non-European), Greece had the highest pharmaceutical spending (expressed as the percentage of gross domestic product) based on data from 2018 to 2021 [67]. An earlier statistical analysis regarding data from 2013 has also revealed high consumption of pharmaceuticals, and more specifically antibiotics, in Greece, with the highest population-weighted consumption data (expressed as defined daily doses per 1000 inhabitants per day) among 30 EU/EEA countries included in the analysis [68].

Besides pharmaceuticals and their metabolites released to the environment after consumption, another entry route via improper disposal of (expired) products is also prominent in Greece. According to a recent report, only 45% of unused and/or expired household pharmaceutical waste is being returned to the pharmacies [69]. In a survey conducted in four European countries, Greece had the highest percentage (>30%) of improper pharmaceutical disposal (flushing them down the drain or throwing them away in garbage bins) and the second-lowest percentage of returning them via the implemented take-back scheme (46%) or being aware of the existence of take-back schemes (<54%) [69]. Environmental degradation and bioaccumulation of pharmaceutical products in Greek waters are of particular concern due to the protected status of several biotopes in the country. For example, Greece was ranked 5th among 27 European countries in terms of the terrestrial portion (in %) of Natura 2000 areas [70] and was reported to have the sixth-highest marine area among 22 EU member states designated as Special Protected Areas within the Natura 2000 network in 2019 [71]. Finally, high levels of pharmaceutical consumption pose a risk not only to ecosystems but also to human health of citizens and tourists, particularly considering that Greece is a popular tourist destination during the summer months. For example, a statistical analysis for 2018 revealed that Greece had the 5th highest net occupancy rates (in %) of bed places in hotels and similar accommodation establishments in the peak summer month (August) among 28 European countries [72].

The objective of the present research is to provide an extensive overview regarding the presence and associated risks of pharmaceuticals in the environment. Particularly, by considering information regarding the consumption of pharmaceuticals, as well as information regarding the monitoring of pharmaceuticals in wastewater and environmental water samples, this research attempts to bridge the gap between the consumption and detection of these emerging micropollutants, and to predict classes of pharmaceuticals that warrant further monitoring in environmental samples. Taking into account the previously described ecotoxicity potential of pharmaceuticals, in correlation with the reported concentrations in WWTP effluent and environmental water samples, a list of priority compounds is generated, which can help guide future legislative efforts on a European level regarding the monitoring of pharmaceuticals in water and wastewater, as well as the effort to develop and apply wastewater treatment techniques, which can target specific micropollutants of high risk.

The present review study demonstrates clear, quantifiable discrepancies between pharmaceutical consumption, environmental occurrence, and ecological risk in Greece. A total of 359 APIs, metabolites, and transformation products were evaluated based on consumption statistics, environmental monitoring data, and risk quotients (RQs), considering the actual concentrations measured in environmental samples and the predicted no-effect concentration (PNEC) for each compound. Of the 241 compounds for which RQs could be calculated, 38 (16%) were categorized as high-risk (RQ ≥ 1), 60 (25%) as medium-risk, and 143 (59%) as low-risk. High-risk substances mainly included nonsteroidal anti-inflammatory drugs (NSAIDs, e.g., diclofenac, ibuprofen), antibiotics (macrolides, fluoroquinolones), synthetic hormones, contrast agents, and triclosan, driven by either high concentrations in wastewater or very low toxicity thresholds. A clear monitoring imbalance was observed. Classes A and B, although widely consumed, are scarcely monitored, while antibiotics, NSAIDs, antidepressants, and analgesics are monitored more frequently than their consumption levels would predict. Metabolites remain under-investigated: most were analyzed only once or twice, and several (e.g., O-desmethylvenlafaxine, norfluoxetine) showed higher risk than their parent compounds. These findings highlight significant gaps between pharmaceutical use, environmental detection, and ecological hazard, underscoring the need for risk-based prioritization in future monitoring frameworks.

## 2. Materials and Methods

### 2.1. Case Study Description: Pharmaceutical Market and Disposal in Greece

According to a recent publication of the Economic and Industrial Research Foundation (IOBE) in cooperation with the Hellenic Association of Pharmaceutical Companies (SfEE) [73], the total health expenditure in Greece for 2020 was EUR 15.7 billion, of which EUR 9.7 billion was public health expenditure, with an average per capita total health expenditure equal to EUR 1468 for 2020, lower than the EU average of EUR 3595. Of the average household’s monthly health expenditure in Greece during 2020, which equals EUR 105.9, 34% corresponds to pharmaceutical products, 31.5% to hospital services, 10.8% to medical services, and 7.6% to therapeutic appliances and equipment. The total expenditure for pharmaceuticals and other medical non-durable goods in Greece was reported at EUR 4.7 billion for 2020. Compared to the EU average, using data from 2019, the per capita public expenditure for pharmaceuticals and other medical non-durable goods was lower in Greece (EUR 226, compared to EUR 319), whereas the private expenditure was higher than the EU average (EUR 172, compared to EUR 132). A total of 106 facilities that produce or import pharmaceutical products were reported, with products being distributed to the population via 10,427 private pharmacies, 128 hospital pharmacies, and 32 pharmacies of the National Organization for the Provision of Health Services (EOPY). Greece has the highest ratio of pharmacies per capita (96 per 100,000 inhabitants in 2020) among the EU27 countries (with an average of 32 per 100,000 inhabitants in 2020 for EU27). Using data from 2020, it was reported that most sales of pharmaceuticals in Greece take place via private pharmacies and wholesalers (i.e., 481 million packages), compared to sales via the hospital or EOPY pharmacies (88 million packages) [73]. The market share of pharmaceuticals for 2020 (in volume) was reported per different patent protection status categories and corresponded to 8.3% for on-patent pharmaceuticals, 33.8% for off-patent, and 35.1% for generics [74]. In Greece, it is legally required to return unused or expired medications, as established by the government take-back schemes, for which collection bins are installed in pharmacies [75,76]. Collected medications are classified as hazardous waste and are treated via incineration [77].

### 2.2. Pharmaceutical Sales and Usage in Greece

Information regarding the annual consumption and sales of pharmaceuticals in Greece from 2010 to 2021 was retrieved from the OECD website [78] as aggregated values for several Anatomical Therapeutic Chemical (ATC) classification system categories. It should be noted that data were not reported for all pharmaceutical classes every year. Data reported by OECD were retrieved by EOPY and do not include over-the-counter (OTC) medication, drugs dispensed in hospitals, and non-reimbursed drugs. Annual data for which all pharmaceutical classes were reported are shown in the Results (Section 3.1).

### 2.3. Presence of APIs, Metabolites, and Transformation Products in the Environment

A thorough screening of the available literature regarding the detection of pharmaceuticals, metabolites, and transformation products in the environment and in WWTPs in Greece was performed on Google Scholar from 1 October to 25 October 2022 using the keywords “pharmaceuticals” or “drugs” and “effluent”, “in wastewater”, “in sewage”, “in water”, “in environment”, and “in drinking water”. Based on this, 55 scientific papers [79,80,81,82,83,84,85,86,87,88,89,90,91,92,93,94,95,96,97,98,99,100,101,102,103,104,105,106,107,108,109,110,111,112,113,114,115,116,117,118,119,120,121,122,123,124,125,126,127,128,129,130,131,132,133] were selected to be included in this paper, providing data on the quantification of 359 APIs, metabolites, and transformation products in municipal and hospital wastewater and sludge samples (influent and effluent), environmental samples (i.e., water and sediment from the sea, lakes, rivers, and stormwater), and bottled water. A detailed overview of the reported findings from the selected publications is given in the Supplementary Information (Table S1) and includes, for each API or metabolite/transformation product, the number of times included in research papers and detected in different (waste)water bodies, as well as the maximum concentration in wastewater effluent and environmental water samples. The reported compounds were also grouped based on the pharmaceutical classes used by the OECD (Section 2.2) in order to correlate the presence of APIs in the environment with their usage, and the analyzed data are reported in the Results section.

### 2.4. Ecotoxicity of APIs, Metabolites and Transformation Products in the Environment 

The final fate of pharmaceuticals and their metabolites and transformation products is to be released to the environment after wastewater treatment. Final receivers of the treated effluents include the sea, rivers, lakes, and groundwater reservoirs. Once released to the environment, these pollutants have been associated with varying degrees of ecotoxicity for several organisms. To assess the environmental risk posed by the identified APIs, metabolites, and transformation products in the samples investigated, the risk quotient (RQ) was calculated as the ratio between the measured environmental concentration (MEC) and the predicted no-effect concentration (PNEC). For each compound, the MEC corresponds to the highest concentration detected in WWTP effluents, hospital effluents, or natural water bodies. The use of the maximum observed MEC represents a conservative, screening-level approach that captures worst-case exposure scenarios, consistent with common practice in preliminary environmental risk assessments [79,100,109,118]. Pharmaceutical concentrations in effluents and receiving waters are highly variable, and the use of maximum values minimizes the risk of underestimating potential ecological effects and aligns with the precautionary principle. However, this approach may overestimate typical environmental risk under average exposure conditions.

To gather information on ecotoxicity for the target compounds, the NORMAN Database System was selected [134]. The NORMAN Network is a European platform providing harmonized, quality-controlled information on contaminants of emerging concern, integrating both experimental and modelled ecotoxicity data. Established originally as an EU-funded initiative, it now operates as a collaborative network supporting the assessment and prioritization of emerging contaminants across Europe. Ecotoxicity values were specifically retrieved from the Substance Database (SusDat) and the Ecotoxicology Database, which compile validated experimental results (from databases such as US EPA ECOTOX, the REACH portal, and regulatory sources) with in silico predictions, and apply the CRED (Criteria for Reporting and Evaluating Ecotoxicity Data) framework to ensure reliability, predominantly calculated for freshwater environments. The lowest PNECs were used as toxicity threshold values for prioritization purposes, as agreed by Europe-wide expert consultation [135]. NORMAN was selected for this assessment because it offers reliable, standardized, and comparable ecotoxicity thresholds suitable for evaluating the environmental hazard of pharmaceuticals on the European scale. It should be noted that NORMAN often provides the most conservative ecotoxicity thresholds compared to values derived from individual studies or tools such as the ECOTOX database, which is advantageous for the prioritization of APIs in this review.

Of the 359 compounds identified in the samples, 340 were found in the NORMAN database, while the remaining 19 compounds were not available and were excluded from the risk assessment. An overview of the reported findings is given in the Supplementary Information and includes the lowest PNEC, the maximum MEC in the wastewater effluent and environmental water samples, and the resulting RQ for each API or metabolite/transformation product (Table S2). The potential risk was categorized as high if RQ ≥ 1, medium if 1 > RQ ≥ 0.1, and low if RQ ≤ 0.1.

## 3. Results and Interpretation

### 3.1. Consumption Data for Pharmaceuticals in Greece

**Figure 2:**
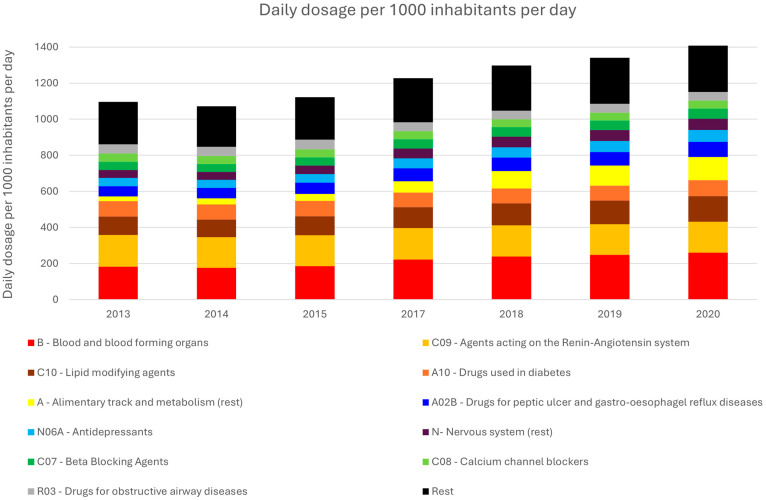
Daily usage of different pharmaceutical classes in Greece from 2010 to 2021.

The daily dosage per 1000 inhabitants for different classes of pharmaceuticals was retrieved from the OECD website for different years (Section 2.2), and the results are shown in Figure 2. As can be observed, an increasing trend in total pharmaceutical consumption is recorded over the years, while the relative percentage of each class to the total sales remains relatively stable. Specifically, the total daily dosage metric has increased from 1096 to 1408 (per 1000 inhabitants) between 2013 and 2020, with **class B** (Blood and blood forming organs) having the highest consumption (18% on average over the displayed years), followed by **class C09** (agents acting on the renin–angiotensin system, 14%), **class C10** (lipid-modifying agents, 10%), **class A10** (drugs used in diabetes, 7%), **class A** (alimentary tract and metabolism), and **class A02B** (drugs for peptic ulcer and gastro-esophageal reflux diseases, both at 6% on average). The only notable deviations over the years can be observed for **class A**, wherein the percentage increases from 2% (2013) to 9% (2020), and **class C09**, decreasing from 26% (2013) to 12% (2020).

### 3.2. Pharmaceuticals Detected in the Environment, Drinking Water, and WWTPs in Greece

**Figure 3:**
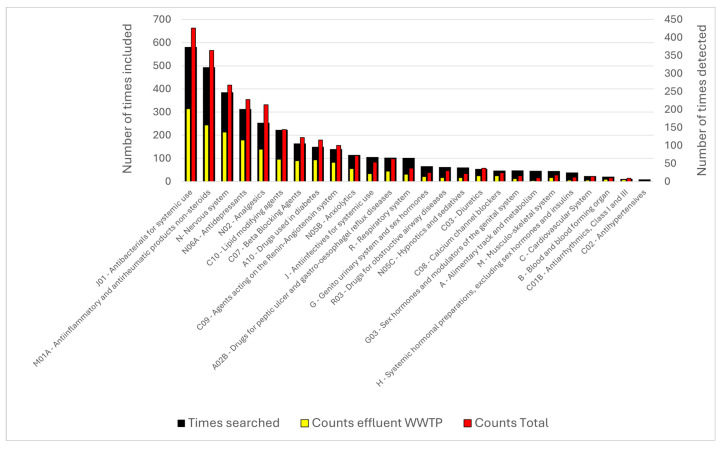
Overview of different APIs, metabolites, and transformation products detected in Greece, organized in therapeutic classes, and sorted in decreasing order based on the number of times included in a research study. Left axis, black bar: number of times a compound was included in the research study. Right axis: number of times a compound was detected (in any water body, red bar), and specifically in wastewater samples (municipal and hospital, yellow bar).

**Figure 4:**
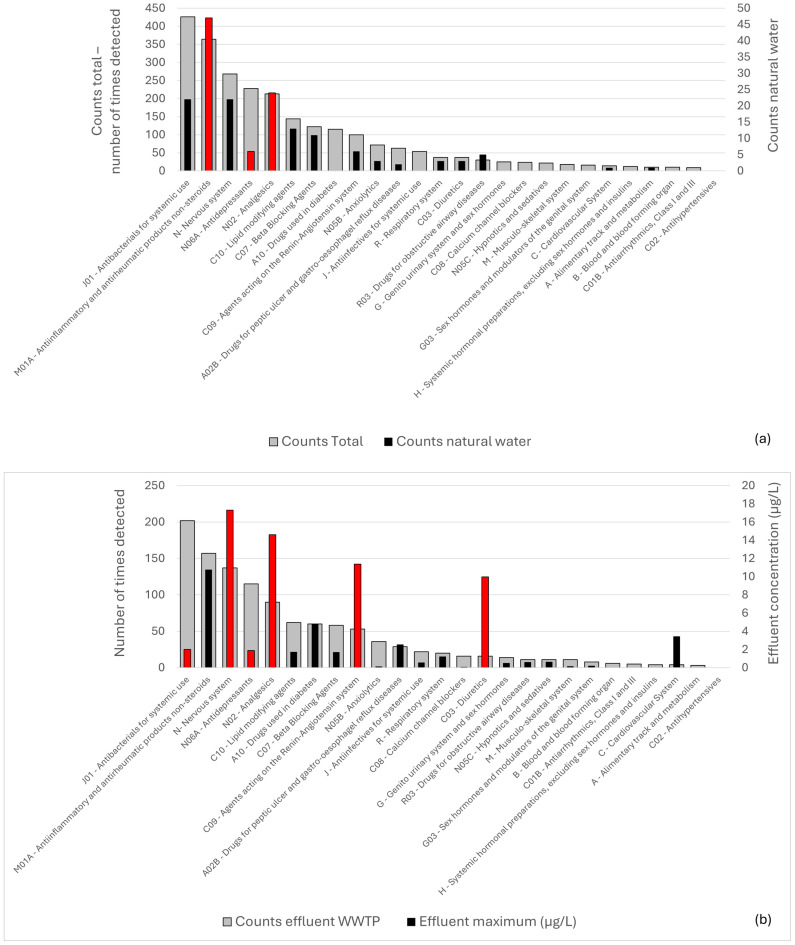
Overview of different APIs, metabolites, and transformation products detected in Greece, organized in therapeutic classes and sorted in decreasing order based on the number of times they were detected in any water sample ((**a**), grey bar, left axis), or the number of times they were detected in WWTP effluent ((**b**), grey bar, left axis). On the right axis, the total counts in natural water samples ((**a**)) and the maximum concentration detected in wastewater are shown (in (**b**)). For each metric, for the data on the right axis, the red bars indicate therapeutic classes where the contribution to the metric on the right axis differs by >5% from the expected proportion based on the left-axis metric.

In total, 359 APIs, metabolites, and transformation products were included in the analysis, based on the literature on pharmaceuticals detected in different water bodies in Greece (Section 2.3). An overview of the times included in each research paper and times detected (in general and in wastewater samples) is shown in Figure 3. Some of the compounds included from the literature do not correspond to a selected therapeutic class for this analysis (based on the categories included in the OECD results for consumption, included in Figure 2). These are excluded from Figure 3 and Figure 4. With some minor exceptions (e.g., number of times diuretics—**class C03** are detected), all three categories follow a similar trend for the different classes. Antibacterials (i.e., **class J01**) are the most commonly researched and detected category, followed by anti-inflammatory and antirheumatic compounds (**class M01A**), drugs used for the nervous system (**class N**), antidepressants (**class N06A**), and analgesics (**class N02**).

The number of times detected in any water body was correlated with the number of times a therapeutic class was detected in natural water bodies. Furthermore, the number of times per therapeutic class detected in WWTP effluent samples was correlated with the maximum concentration detected in effluents, and the results are shown in Figure 4a and b, respectively. Here, a few outliers were observed, wherein the number of times detected in nature (Figure 4a) or the maximum concentration (Figure 4b) was higher or lower than expected, compared to the corresponding metric on the y-axis for a certain therapeutic class (i.e., number of times detected in any water sample in Figure 4a, or number of times compounds were detected in wastewater effluents in Figure 4b). The contribution of each class to the total measurements per metric (i.e., times detected, concentration) was calculated and compared among the x-axis and y-axis metrics in Figure 4 to identify outliers (i.e., classes that differed more than 5% in percentage between metrics), which are displayed in red in the figure.

As can be observed in Figure 4a, anti-inflammatory and antirheumatic products (**class M01A**) and analgesics (**class N02**) were detected more times, while antidepressants (**class N06A**) were detected fewer times in natural waters than expected, assuming a proportional relationship to the overall number of times compounds are detected in the included literature. Within **class M01A**, the APIs mainly detected in natural waters are diclofenac, naproxen, ibuprofen, and ketoprofen, all of which are NSAID medications that are available over the counter in Greece. For **class N02**, the primary compounds detected in natural waters are paracetamol, salicylic acid, and phenazone, i.e., common analgesic and antipyretic medications that are also available over the counter in Greece. Likely, the frequent detection of these compounds in natural waters near anthropogenic activities is due to their widespread use.

In Figure 4b, medications for the nervous system (**class N**), analgesics (**class N02**), hypertension, and heart failure (i.e., agents acting on the renin–angiotensin system, **class C09**), and diuretics (**class C03**) have higher concentrations, while antibacterials (**class J01**) and antidepressants (**class N06A**) have lower concentrations than expected, assuming a proportional relationship to the overall amount of times compounds are detected in wastewater effluent samples. For analgesics, as discussed in the previous paragraph, the class includes several popular over-the-counter products, which are not included in the OECD list of consumption data, likely explaining the deviation between the two metrics. For **class N**, the deviation is primarily due to **valproic acid**, which is detected only nine times but reaches a maximum reported concentration in effluent samples of 17 μg/L (highest recorded among all examined APIs investigated in this study), compared to, for example, the component with the second highest concentration in this class (carbamazepine, at 2.2 μg/L), which is detected 71 times. Among the compounds contributing to the high wastewater concentration for **class C09**, **valsartan** has the second-highest concentration among all APIs investigated in this study (11 μg/L), and **irbesartan** has the seventh-highest (around 7.5 μg/L), while the total number of times detected does not exceed 30 for either compound, compared to, for example, ibuprofen (class M01A, 10.7 μg/L, third-highest concentration in wastewater, with 59 total times detected). Similarly, for **class C03**, **furosemide** is primarily responsible for this trend, as it has by far the highest effluent concentration among the five APIs included (10 μg/L, fifth-highest concentration in wastewater) and only 24 total counts for detection in any water sample.

### 3.3. Expected Concentrations of Pharmaceutical-Related Pollutants Based on Consumption—Is There a Gap Between Consumption and Detection?

**Figure 5:**
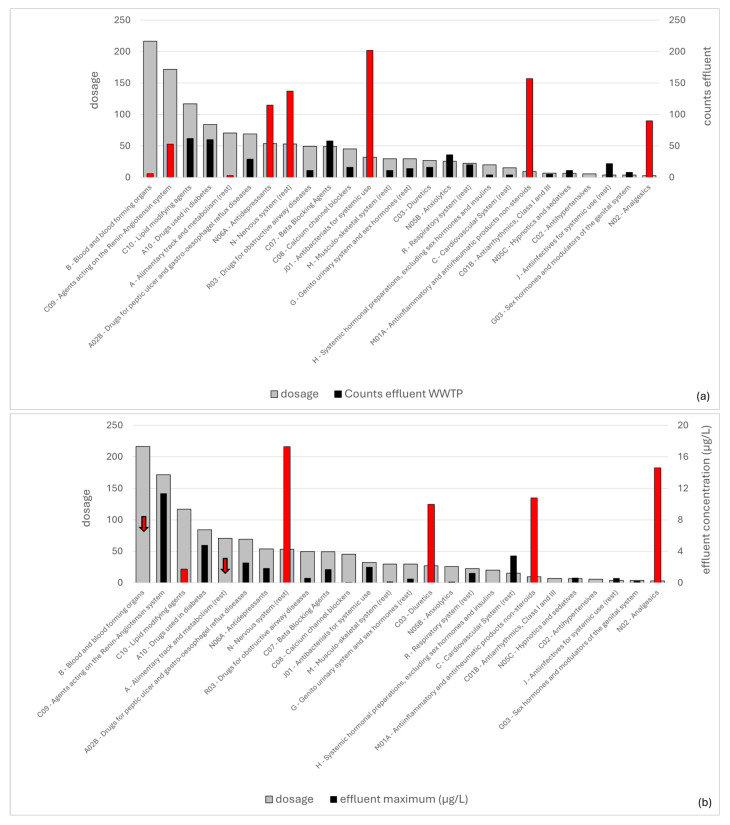
Overview of different APIs organized in therapeutic classes and sorted in decreasing order based on the daily dosage per 1000 inhabitants (data from OECD, grey bar, left axis). On the right axis, different metrics are shown based on APIs, metabolites, and transformation products researched in Greek water bodies (black bars). For the data on the right axis, the red bars (or red arrows, when red bars are too small to be visible) indicate therapeutic classes where the contribution to the metric on the right axis differs by >5% from the expected proportion based on the left-axis metric. (**a**): total counts in WWTP effluent samples (municipal and hospital). (**b**): maximum concentration in effluents of WWTPs.

The correlation between the number of times pharmaceuticals were detected in effluents or their maximum effluent concentration per therapeutic class and the daily dosage data retrieved from the OECD was investigated, and the results are presented in Figure 5. Several therapeutic classes were identified as outliers when correlated with the corresponding dosage, and these are indicated in red in the figure. To identify outliers (i.e., classes that differed more than 5% in percentage between metrics), the contribution of each class to the total measurements per metric (i.e., dosage, times detected, concentration) was calculated and compared among the x-axis and y-axis metrics.

Several classes were identified as common outliers between the times detected in WWTP effluents (Figure 5a) or the effluent concentrations (Figure 5b) compared to the corresponding predicted metric assuming a correlation with the dosage for the class. Specifically, **class B** (blood and blood-forming products) and **class A** (alimentary tract and metabolism) were both under-detected, whereas **class N** (nervous system), **class M01A** (anti-inflammatory and antirheumatic products), and **class N02** (analgesics) were all over-detected, when compared to the dosage for each class. Some of the classes identified as outliers follow a different trend between the counts and the concentration in effluent samples. Specifically, **class N06A** (antidepressants) and **class J01** (antibacterials) were over-detected in terms of counts but not in terms of effluent concentration, while **class C03** (diuretics) was over-detected only in terms of concentration. On the contrary, **class C09** (agents acting on the renin–angiotensin system) and **class C10** (lipid-modifying agents) were under-detected only in terms of counts and concentration, respectively.

Medications in **class B** have the highest daily dosage among the API classes in the OECD dataset and include 71 APIs in the ClinPGx Database [135] (excluding salicylic acid and sulfamethizole, which have been included in other API classes here). Nevertheless, only four APIs have been included from that therapeutic class in the literature researching pharmaceuticals in different water bodies, namely clopidogrel, rivaroxaban, tranexamic acid, and warfarin. Likely, the under-detection of this class is due to compounds being prescribed and consumed in Greece, while not included in literature studies estimating concentrations in the environment and WWTPs. Similarly, for APIs in **class A** [136], 203 APIs belong to this class, which is recalculated to 176 (excluding substances included in other classes), while only six APIs have been included in literature testing WWTP and natural waters for pharmaceuticals in Greece. For **class C09**, 10 APIs have been investigated in WWTP and natural water measurements in Greece, compared to the total of 23 APIs included in this class ([136], corrected to exclude compounds from other classes). Similarly, for **class C10**, 9 APIs have been investigated, compared to the total of 25 APIs included in the class ([136], corrected to exclude compounds from other classes).

Overall, regarding API classes that are under-represented in the literature, either in terms of wastewater counts or concentration, two groups can be observed. For **class A and class B medications**, relatively few compounds have been investigated compared to the total number of APIs included in the respective classes. For compounds retrieved from the ClinPGx database, a screening of PNEC values is performed to identify potential priority compounds with high ecotoxicity potential that are currently not considered for monitoring. On the other hand, **classes C09 and C10** are better represented in monitoring campaigns, comparing the number of APIs investigated and the total number of APIs in each class. **Class C09**, taking into account both the higher concentration (Figure 4b) and the higher daily consumption (Figure 5a), compared to the number of times detected in wastewater, contains potential priority compounds for monitoring. For **class C10**, the lower-than-expected concentration compared to the dosage (Figure 5a) may indicate the metabolic breakdown or transformation of APIs, or that the APIs selected for monitoring do not fully represent the entire class in terms of consumption in Greece. Therefore, for this class, other compounds from the ClinPGx Database are screened in terms of PNEC to identify potential hotspots that are currently excluded from monitoring in natural waters and wastewater.

Considering the API classes that were over-detected, either in terms of counts or in terms of concentration, compared to the daily dosage data, different trends can be observed. For **class M01A**, 20 APIs have been investigated in water and wastewater, compared to the 35 APIs included in the class [136]. For **class N02**, 16 APIs (or their corresponding metabolites) have been investigated, compared to the 47 APIs included in the class [136]. As also discussed in Section 3.2, both classes contain several common OTC medications, and therefore, one possible explanation for the trend observed in Figure 5 is that daily dosage data are underestimated, compared to pharmaceutical classes that require a prescription and are therefore easier to track in terms of sales. Importantly, the OECD consumption data used here do not include OTC medication, drugs dispensed in hospitals, or non-reimbursed drugs. For **class N06A**, 19 APIs have been investigated, out of the 38 APIs included in the class [136], while for **class C03**, only 5 APIs have been investigated, compared to the 22 APIs included in the class [136]. As was also discussed in Section 3.2, both **class N06A and J01** have been investigated (and counted) disproportionately many times, compared to their effluent concentration (Figure 4b), and this is also the case when compared to the daily dosage (Figure 5a), while **class N06A** is also well represented in terms of number of APIs investigated compared to the total APIs in the class. The reason why these classes are over-represented in the literature could be related to their high consumption and ecotoxicity potential [137] or the scope of the literature included in this analysis, which contained six papers reporting measurements at a psychiatric hospital [95,96,97,98,99,100]. Whether the research effort of quantifying these compounds in nature and effluent samples is warranted should be considered in terms of their ecotoxicity potential. Similarly, for **class C03**, the disproportionately high effluent concentration, compared to the daily dosage and effluent counts, should be taken into account during the ecotoxicity assessment of different APIs, particularly for **furosemide**, which is the primary compound responsible for the high effluent concentration of class C03.

Overall, based on the analysis in Section 3.2 and Section 3.3, the following classes are identified as potential priority groups to examine due to their high counts, effluent concentration, daily dosage, and representation of the total number of APIs per class in the number of APIs selected for monitoring: **class N02**—potential hotspot due to high effluent concentration; **class C03**—potential hotspot due to high effluent concentrations, particularly **furosemide**; **class N**—potential hotspot due to high concentration, particularly **valproic acid**; **class C09**—potential hotspots due to high daily dosage and effluent concentration; **classes A**, **B**, **C10**—potential hotspots due to high dosage and being under-represented in monitoring; and **classes J01**, **N06A**, **M01A**—over-represented in monitoring and may need to be reconsidered as hotspots.

### 3.4. Ecotoxicity Assessment for Detected Compounds

**Figure 6:**
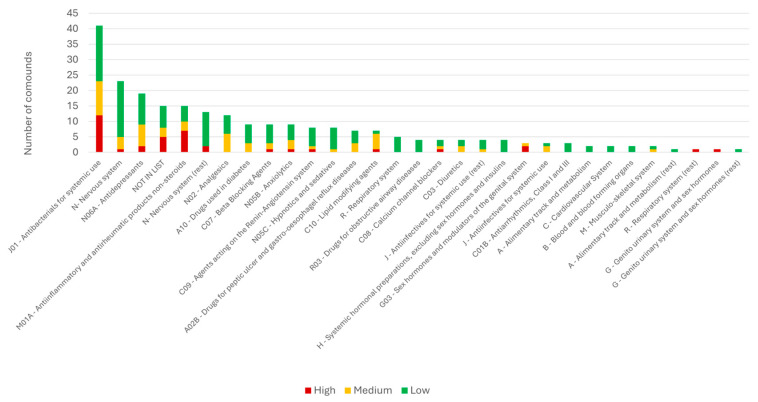
Distribution of RQ categories (low, medium, high) across therapeutic pharmaceutical classes. The height of each bar indicates the total number of substances in each class, while colors represent the proportion assigned to each risk category.

A total of 359 APIs, metabolites, and transformation products were considered in the ecotoxicological assessment. For 340 of these substances, a freshwater PNEC could be retrieved from the NORMAN Ecotoxicology Database, whereas 19 substances were not listed and were therefore excluded from the quantitative risk assessment. These substances are mainly metabolites across several pharmaceutical classes, with most of them (6 out of 19) falling into calcium channel blockers (C09 class). For each compound, the highest concentration measured in Greek WWTP effluents or natural waters was used as the MEC, and the corresponding RQ was calculated (Section 2.4). For 77 substances that were analyzed in effluent samples but never detected, no RQ could be determined because no measurable concentration above the analytical limit of detection (LOD) was available. These undetected substances span several therapeutic classes, including J01, N, N02, N06, G, and M01A, as well as compounds categorized outside the OECD classes. A further 22 substances, including compounds from the J class and “NOT IN LIST”, had been analyzed only in influent samples, and therefore no RQ was presented due to the focus on effluent samples. Overall, RQs were successfully derived for 241 compounds, whereas 118 substances could not be assigned an RQ due to missing PNEC values or the absence of detectable MECs. An overview of these results is provided in the Supplementary Material (Table S2). Among the 241 compounds with available RQs, 38 substances (16%) fell into the high-risk category (RQ ≥ 1), 60 substances (25%) into the medium-risk category (0.1 ≤ RQ < 1), and 143 substances (59%) into the low-risk category (RQ < 0.1). Figure 6 illustrates the distribution of RQ categories across therapeutic classes.

**Figure 7:**
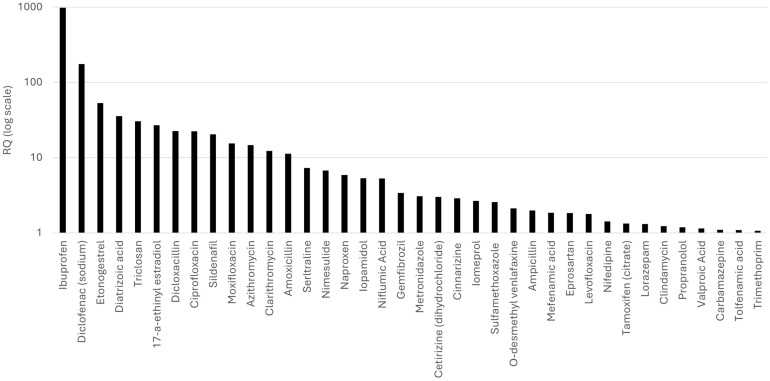
Highest-risk pharmaceuticals in WWTP effluents based on RQ values (log scale).

The classes contributing the highest proportion of medium- and high-risk substances were antibacterials (**class J01**), anti-inflammatory and antirheumatic products (**class M01A**), antidepressants (**class N06A**), and a group of compounds outside the OECD classes (“NOT IN LIST”). To further analyze their contribution, the individual substances driving the risk within these classes are discussed, while Figure 7 presents the 38 substances with the highest RQ values of the samples investigated.

Antibacterials (**class J01**) contributed the largest number (12 in total) of high-risk compounds (Figure 7), including **dicloxacillin**, **ciprofloxacin**, **moxifloxacin**, **azithromycin**, **clarithromycin**, **amoxicillin,** and **metronidazole** (antibacterials and antibiotics). Many of these antibiotics did not necessarily appear at the highest measured concentrations in WWTP effluents or water samples (up to 2 μg/L), but their low PNECs (0.005–0.13 μg/L) yielded high RQs (up to 22) even at moderate MEC values. This class also contained the highest number of medium-risk compounds (11 substances), including ofloxacin, tetracycline, norfloxacin, and erythromycin. A similar pattern was observed for anti-inflammatory and antirheumatic drugs (**class M01A**), where seven of the fifteen evaluated NSAIDs exhibited high RQs. **Ibuprofen** and **diclofenac** constitute the two most toxic compounds in the entire dataset (Figure 7), combining high effluent concentrations (up to 10 µg/L) with low PNECs (0.01–0.04 µg/L). Nimesulide, naproxen, and niflumic acid also reached high effluent concentrations (up to 10 µg/L), but their PNECs were comparatively higher (0.14–1.7 µg/L), resulting in lower but still significant positions within the high-risk rank.

Several substances classified as “NOT IN LIST” also appeared among the highest-risk chemicals. These include the radiocontrast agent **diatrizoic acid**, the disinfectant **triclosan**, and the contrast medium **iopamidol**. The first two compounds appear in the top five highest-risk compounds. Their elevated RQs are attributed to both high effluent concentrations in Greece (especially for triclosan) and to relatively low PNECs. Although fewer in number, hormonal pharmaceuticals (**class G03**) were among the most hazardous substances assessed. **17α-ethinyl estradiol** and **etonogestrel** reached some of the highest RQs in the dataset (Figure 7), despite MECs often in the low or sub-ng/L range, reflecting their extremely low PNECs and potent risk effects. Beyond these major groups, several additional compounds complete the list of the top 20 highest-risk pharmaceuticals. These include **sildenafil** (**class G**), **sertraline** (**class N06A**), and **gemfibrozil** (**class C10**). The G and C10 classes each include only one high-risk compound, whereas N06A contains two (sertraline and O-desmethylvenlafaxine). The broader **N06A class** also includes seven medium-risk substances (out of 19), such as doxepin and venlafaxine, whose RQs approach 1 even though environmental concentrations recorded in Greece are comparatively low (Figure 4b).

On the other hand, some therapeutic categories contributed predominantly low-risk substances, notably **class N** (nervous system) and **class J01**, despite the latter’s substantial contribution to the high-risk group. Both classes contained 18 low-risk compounds each. Low-risk nervous system pharmaceuticals include prilocaine, rivastigmine, sulpiride, and oxcarbazepine, while low-risk antibacterials include sulfonamides (sulfathiazole, sulfamethizole), clindamycin sulfoxide, and desmethyl-clarithromycin. It is worth noting that within class N, **valproic acid** exhibited the highest MEC in the entire dataset (Figure 4b). However, its PNEC is not particularly low, and therefore, its RQ does not place it within the top 20 most toxic compounds, although it still falls within the high-risk category (Figure 7). The “nervous system (rest)” subgroup similarly contains mainly low-risk substances, including 10-hydroxycarbazepine, 9-OH risperidone, amantadine, and amphetamine.

Overall, the ecotoxicity assessment indicates that a subset of the investigated pharmaceuticals in Greece are likely to pose a high risk to aquatic ecosystems at present environmental levels. These high-risk compounds include several widely used NSAIDs, antibiotics, and sex hormones, as the dominant drivers of ecological risk in Greek aquatic environments.

### 3.5. Correlations Between Ecotoxicity Assessment, Dosage, and Detection in the Environment

To better contextualize the ecotoxicological patterns described in Section 3.4, an integrated assessment was performed examining how pharmaceutical consumption, monitoring intensity, and detection and ecotoxicity relate. The combined interpretation highlights areas of agreement between environmental occurrence and ecotoxicological relevance, but also potential mismatches that influence prioritization of pharmaceuticals in Greece.

**Figure 8:**
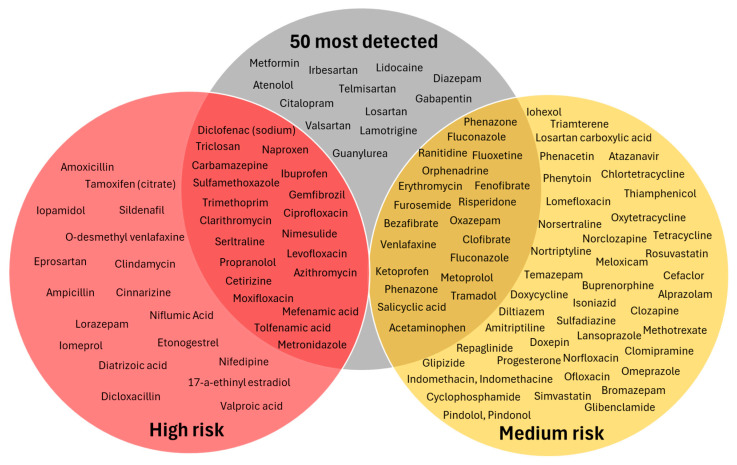
Venn diagram for the overlap between the 50 most frequently detected compounds (gray) and the pharmaceuticals categorized by environmental risk levels (high risk in red, medium risk in yellow).

Figure 8 illustrates the overlap between the fifty most frequently detected compounds in WWTP effluents and natural water samples, and those classified as high- or medium-risk. A large proportion of both risk categories appears among the most frequently detected substances, with a substantial number of compounds falling within the intersection of frequent detection and elevated risk. Notably, 18 medium-risk compounds are included among the top 50 most detected chemicals, indicating that many pharmaceuticals with moderate ecotoxicological concern are also widely present. Furthermore, the proportion of high-risk compounds represented in the top 50 is considerable. Although the total number of high-risk compounds in the dataset is relatively small (38 in total), 20 of them appear among the most frequently detected pharmaceuticals. Among these, 12 compounds are ranked within the top 20 highest-risk substances (RQ up to 3.4), as discussed in Section 3.4. These include several NSAIDs (i.e., **class M01A**, ibuprofen, diclofenac, nimesulide, and naproxen), antibiotics (i.e., **class J01**, ciprofloxacin, moxifloxacin, azithromycin, clarithromycin, and metronidazole), a biocide (**triclosan**), a psychiatric medication (**class N06A**, sertraline), and a lipid regulator (**class C10**, gemfibrozil). These compounds are detected between 8 and 57 times, with relatively high detection-to-search ratios. Conversely, fourteen pharmaceuticals appear frequently in effluent samples but fall into the low-risk category. These include, among others, agents acting on the renin angiotensin system (i.e., **class C09**, such as telmisartan, valsartan, irbesartan, and losartan). Notably, **valsartan** and **irbesartan** exhibit some of the highest effluent concentrations in the dataset (Figure 4b), yet their RQs remain low, demonstrating that frequent detection alone does not necessarily correspond to elevated ecological risk. Their high detection frequency is more likely explained by widespread consumption, consistent with the high dosage observed for the **C09 class** (Figure 5b), combined with routine analytical inclusion.

**Figure 9:**
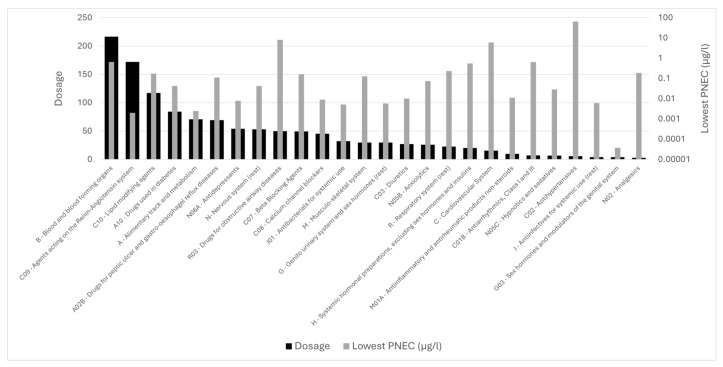
Comparison of daily dosage (black bars) and the lowest PNEC value (grey bars) among compounds within each pharmaceutical class.

Figure 9 compares the daily dosage of each pharmaceutical class with the lowest PNEC value identified within that class. The lowest PNECs are observed for the hormonal pharmaceuticals (**class G03**), reaching values down to 0.037 ng/L, which are several orders of magnitude lower than those of any other class, followed by classes such as **C09**, **A10**, **J01,** and **J**, which present moderate PNECs. In contrast, most highly consumed classes, such as blood and blood-forming organs (**class B**), lipid-modifying agents (**class C10**), and antidiabetics (**class A10**), exhibit comparatively higher PNECs. When considered together with their generally low MECs (Figure 5b), these classes tend to fall within the low to medium ecotoxicity range. Agents acting on the renin–angiotensin system (**class C09**) also show moderate PNECs, and despite some compounds exhibiting elevated MECs, the majority of substances in this class (6 out of 8) remain in the low-risk category. In contrast, even the low environmental concentrations of hormonal pharmaceuticals (**class G03**) result in high RQs because of their exceptionally low PNECs. This illustrates the strong influence of intrinsic toxicity on overall risk characterization, independent of dosage. Overall, this comparison shows that consumption alone is not indicative of ecotoxicological relevance. Because dosage and MEC patterns may vary across classes (as discussed in Section 3.3), meaningful evaluation of environmental impact requires consideration of both MEC and PNEC.

**Figure 10:**
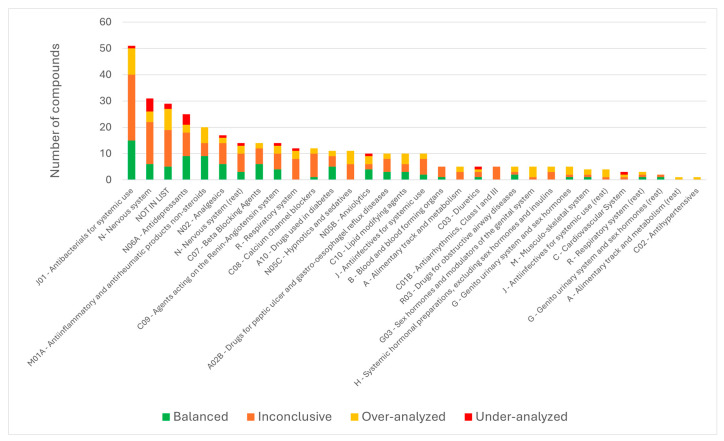
Distribution of pharmaceuticals across ATC classes according to monitoring evaluation status. Bars represent the total number of compounds within each ATC category, categorized into balanced, inconclusive, over-analyzed, and under-analyzed substances.

To evaluate the alignment between monitoring effort and environmental occurrence, a detection ratio (times detected divided by times searched in effluent) was calculated for each compound (as shown in the Supplementary Information, Table S2). Pharmaceuticals showing low detection ratios despite extensive monitoring were classified as over-analyzed, whereas compounds with comparatively high detection ratios but limited monitoring coverage were considered under-analyzed. Substances analyzed only once or twice were classified as inconclusive, even where very high detection ratios were observed. The results are presented in Figure 10.

Across most classes, a large proportion of compounds fall into the inconclusive category, indicating that many APIs were searched in only one or two studies or detected infrequently to establish reliable monitoring trends. This is particularly evident in substances in the nervous system (**class N**), antidepressants (**class N06A**), and antibacterials (**class J01**), where a substantial number of compounds have been analyzed sporadically. Several classes also present notable proportions of over-analyzed substances, such as **classes J01, M01A, and N05C** and compounds outside the OECD list. These APIs are frequently searched despite low or inconsistent detections, likely due to their historical prominence or analytical convenience. While such monitoring reflects established analytical focus, it contributes little to improving environmental risk understanding, as many of these substances are associated with low RQs and low environmental concentrations. In contrast, a number of classes include under-analyzed compounds, substances with comparatively high detection ratios but limited monitoring effort. Notably, these appear in the nervous system (**class N**) and antidepressant (**class N06A**) classes. For these, the available detections suggest environmental relevance, yet the low frequency of analytical inclusion indicates insufficient coverage. Many of these under-analyzed compounds, however, exhibit low RQs and fall within the low-risk category. For those substances, additional monitoring is not an immediate priority.

**Figure 11:**
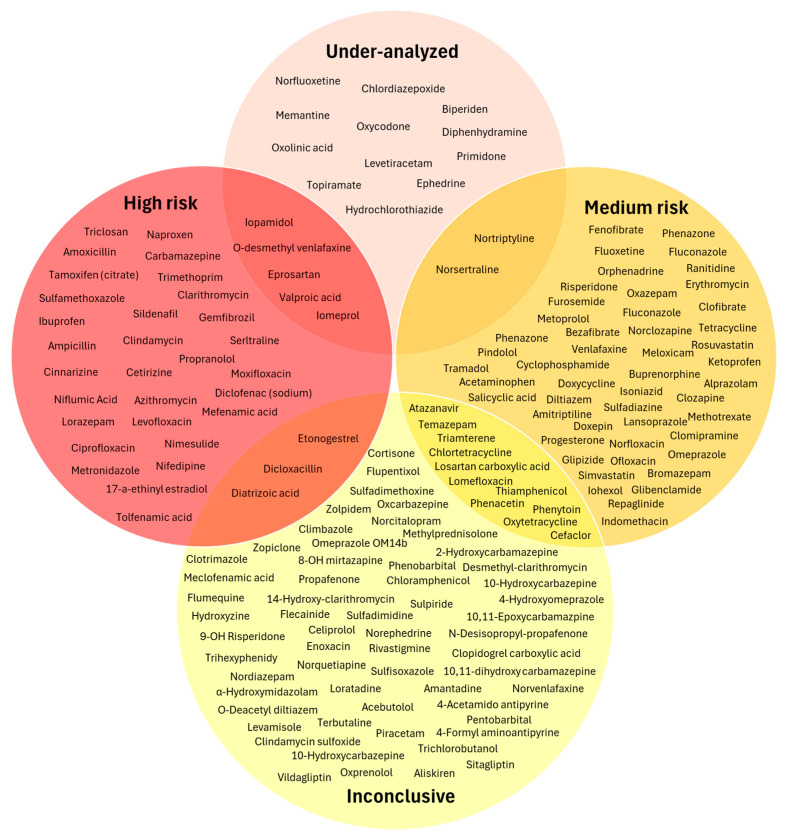
Venn diagram of ecotoxicological risk categories (high, medium) and monitoring coverage (under-analyzed and inconclusive).

The combined interpretation of monitoring coverage (Figure 10) and ecotoxicological risk (as shown in Figure 11) allows identification of APIs that should be prioritized for future monitoring. Only a small subset of under-analyzed compounds also exhibit high or medium RQs, making them environmentally relevant yet insufficiently monitored. These include **O-desmethylvenlafaxine**, **iopamidol**, **eprosartan**, **iomeprol**, and **valproic acid** (high-risk category), as well as **norsertraline** and **nortriptyline** in the medium-risk category. The inconclusive group also contains twelve medium-risk substances, including triamterene and phenacetin, both exhibiting RQs close to 1. Targeted monitoring of these APIs is therefore recommended to clarify their environmental occurrence and refine their risk characterization. However, the majority of under-analyzed or inconclusive compounds fall into the low-risk category, indicating that despite limited analytical inclusion, their ecotoxicological relevance is likely minor. These substances do not require immediate prioritization under current environmental conditions, but further analysis for validation purposes should be considered.

In addition, several classes contain subsets of balanced compounds for which monitoring effort is appropriately aligned with environmental occurrence. Examples include selected APIs in the antibacterials (**class J01**), anti-inflammatory drugs (**class M01A**), and antidepressants (**class N06A**), where the most frequently detected compounds are also routinely searched.

Overall, the integration of monitoring coverage and ecotoxicological risk indicates further improvement is needed to capture actual environmental relevance. While many APIs are over-represented in monitoring campaigns despite low risk (37 out of 62 compounds), several environmentally significant compounds remain under-monitored. Future monitoring strategies should therefore adopt a risk-based approach that prioritizes APIs that are both ecotoxicologically relevant and insufficiently monitored to ensure that substances with the greatest ecological impact are adequately assessed.

**Figure 12:**
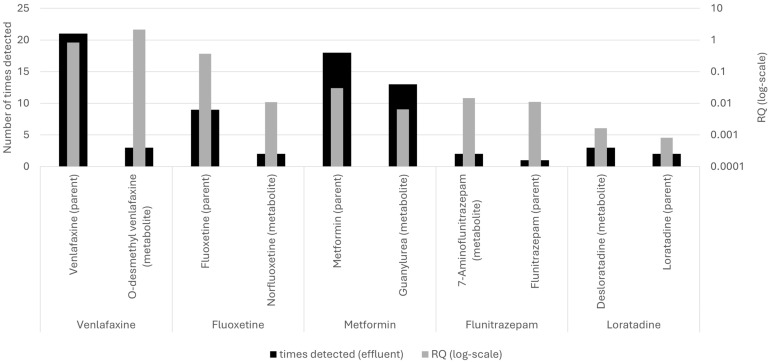
Detection frequency and risk quotient (RQ) of selected API–metabolite pairs detected in WWTP effluents. Bars represent the number of detections per compound, while the line shows RQ values (log scale).

Due to the (metabolic) transformation of many pharmaceuticals in humans and within wastewater systems, it is essential to consider not only parent APIs but also their metabolites when evaluating environmental monitoring data. Although 49 metabolites and transformation products were included in the dataset (Supplementary Material, Table S1), most were analyzed only once or twice in effluent samples (44 compounds) and thus fall into the “inconclusive” category based on the monitoring assessment. This limited analytical coverage prevents a robust evaluation of their environmental occurrence and associated risk. For 19 of those which fell into this category (only a single search and detection), no PNEC value was available, and they were excluded from the quantitative risk assessment, as discussed in Section 3.4. Figure 12 summarizes the detection frequency and RQ values for selected parent APIs and their corresponding metabolites that were among the more frequently monitored in effluent samples. Overall, the figure highlights that parent APIs are generally searched for and detected more often than their metabolites, yet metabolites may contribute disproportionately to ecological risk.

Among them, **O-desmethylvenlafaxine**, the primary metabolite of venlafaxine, shows the strongest environmental significance, combining a limited detection frequency with a high RQ. Although its parent API is present several times, the metabolite is under-analyzed and needs further investigation due to its potential high risk. A similar pattern is observed for losartan and its metabolite losartan carboxylic acid, with both compounds detected in effluents, and the metabolite exhibiting a noticeably higher RQ but an extremely limited search (only one time), not leading to robust conclusions. In contrast, metabolites such as norfluoxetine (from fluoxetine) and desloratadine (from loratadine) show low detection frequencies and low RQs. Desloratadine is an example of an over-analyzed compound, being searched in eight effluent samples but only detected in three. Within the venlafaxine group, venlafaxine (parent) shows the highest detection frequency among the compounds displayed (20 detections) and falls within the medium-risk category, whereas its primary metabolite O-desmethylvenlafaxine exhibits a higher RQ, placing it in the high-risk category despite being monitored fewer times. This indicates that the metabolite may pose greater ecological concern than the parent compound but remains comparatively under-analyzed. A similar pattern appears for the fluoxetine group, where fluoxetine (parent) is detected frequently, whereas norfluoxetine shows a lower detection frequency but a higher RQ. This again suggests enhanced toxicological relevance of the metabolite relative to the parent API.

In contrast to these patterns, metformin and its transformation product guanylurea show the opposite trend. Metformin (parent) is detected more frequently and displays a higher RQ than its transformation product, in agreement with previous findings that guanylurea is typically less toxic and less environmentally persistent than the parent API, and both remain in the low-risk category. For the flunitrazepam group, both the parent compound and its metabolite 7-aminoflunitrazepam show low detection frequencies and fall within the low-risk category. Both compounds are classified as over-analyzed, having been searched several times but detected infrequently. This suggests that neither compound currently represents a major ecotoxicological concern, although their repeated analytical inclusion (up to 5 times) indicates continued interest in this therapeutic group. For flunitrazepam, the metabolite 7-aminoflunitrazepam is detected less often than flunitrazepam but has a noticeably higher RQ, indicating closer attention due to its potential increased ecotoxicity risk. Finally, loratadine and its metabolite desloratadine both show low detection frequencies and low RQs. Desloratadine is an example of an over-analyzed compound, as it was searched several times but detected rarely (3 out of 8), suggesting limited environmental relevance under current conditions. Overall, Figure 12 demonstrates that metabolites can differ from their parent compounds in both environmental occurrence and toxicity. In several cases (e.g., O-desmethylvenlafaxine, norfluoxetine, 7-aminoflunitrazepam), metabolites show higher RQs but lower monitoring effort, indicating that current monitoring practices underestimate the potential contribution of transformation products to overall ecological risk.

## 4. Discussion

Taking into account the consumption of different pharmaceutical classes (Section 3.1), the frequency of monitoring and detection in (waste)water samples (Section 3.2), the correlation between these two metrics (Section 3.3), the reported ecotoxicity potential for the different pharmaceuticals investigated (Section 3.4), and the correlation of ecotoxicity with the consumption and monitoring (Section 3.5), several potential hotspots were identified, i.e., compounds or pharmaceutical classes that ought to be examined as priority targets for environmental monitoring. The identified substances and the criteria for their inclusion as priority substances are summarized in Table 1 and are further discussed in this section.

Three classes were proposed as potential hotspots based on the high concentrations detected in wastewater effluent samples (Section 3.3), and these are **class N02**, **class C03** (particularly **furosemide**), and **class N** (particularly **valproic acid**). Nevertheless, despite the high concentration, classes N02 and C03 do not contain any high-risk compounds based on the calculated RQ values (Figure 6), with furosemide (the main compound causing the observed trends for class C03) itself classified as medium-risk. On the contrary, class N contains both high-risk and medium-risk compounds (Figure 6), with valproic acid in particular being classified as a high-risk compound in terms of ecotoxicity, while also including a significant percentage of inconclusively monitored and under-analyzed compounds (Figure 10). Based on these findings, **class N** is classified as a hotspot class. It should be noted that **class N02** also contains a relatively high percentage of inconclusively monitored compounds, while at the same time being relatively under-represented in monitoring literature, compared to the total number of compounds that belong to this class. Therefore, to conclusively propose any of the compounds of this class as potential hotspots, additional analysis of PNEC values ought to be performed on compounds that belong to this class but are currently not considered in environmental monitoring, as these could be present but not yet identified in natural water and wastewater.

**Class C09** was proposed as a potential hotspot due to the high consumption in Greece and the high concentrations recorded in wastewater effluent samples (Section 3.3). While the majority of compounds in this class are characterized as low-risk in terms of ecotoxicity (Figure 6), some medium- and high-risk compounds are also included in class C09. Considering that this class is well represented in monitoring campaigns compared to the total number of compounds belonging to the class, no additional effort is recommended based on the analysis performed in the present work. On the contrary, based on the analysis presented in Section 3.2 and Section 3.3, three classes were identified as over-represented in the monitoring campaigns, despite their relatively low consumption and effluent water concentration, namely **classes J01, N06A, and M01A**. Nevertheless, the significant percentage of high-risk compounds in these classes (Figure 6), as well as the high percentage of under-analyzed and inconclusive compounds for classes J01 and N06A (Figure 10), indicates that all three should be considered hotspot classes.

Finally, **class A, class B, and class C10** were proposed as potential hotspots (Section 3.3) due to their significant consumption compared to other examined classes, and the fact that they are under-represented in monitoring campaigns, both in terms of monitoring effort (i.e., counts in monitoring literature compared to the high dosage) and in terms of representation of the class (i.e., the total number of compounds belonging to the class compared to the number of different compounds researched in (waste)water). For classes A and B, all the examined compounds were shown to have low risk, while for class C10, the majority of compounds fall into the medium-risk category in terms of ecotoxicity (Figure 6). Nevertheless, when additional compounds for these categories are examined in terms of toxicity, besides the compounds retrieved from monitoring literature, potential hotspots are identified that have significantly lower PNECs compared to the compounds identified in monitoring literature. Specifically, for **class A**, when sorting all compounds from the ClinPGx Database in decreasing order of PNEC, the compound with the lowest PNEC (rifaximin, 0.0025 μg/L, no RQ calculated due to a lack of data on effluent concentration) is, in fact, included in the monitoring campaigns, while the next compound investigated in (waste)water is ondansetron, ranked 55th in terms of PNEC at over 0.99 μg/L. Clearly, several compounds belonging to this class could be potential hotspots based on their PNEC values but may not have been identified as such yet due to lack of inclusion in monitoring campaigns. Similarly, for **class B**, when all compounds from the ClinPGx Database are sorted based on increasing order of PNEC, warfarin is the first compound included in monitoring campaigns at a PNEC concentration of 1.2 μg/L, while 17 compounds have lower PNEC values, ranging between 0.0018 (for dabigatran etexilate and lusutrombopag) and 0.79 μg/L. For **class C10**, fluvastatin has a PNEC value 128 times and 38 times higher, respectively, than lomitapide and probucol (i.e., the compounds belonging to this class that have the lowest retrieved PNEC values). For all three classes, the results indicate that potential hotspots exist, and therefore, it is recommended to include these classes on the priority list and perform screening on wastewater effluent samples for the individual compounds with the lowest retrieved PNEC values.

Besides the potential hotspot groups and compounds identified based on the monitoring and consumption data (i.e., Section 3.2 and Section 3.3), several priority groups and compounds were also proposed, considering the ecotoxicity of different components (Section 3.4 and Section 3.5). For example, the compounds presented in Figure 7, which fall under the high-risk category with a calculated RQ > 1, ought to be considered as potential hotspots, as their measured concentration in the environment significantly exceeds their corresponding PNEC values. While the measured environmental concentration is an important metric to consider, it does not necessarily provide a meaningful metric by itself, considering that an individual high concentration value may have been due to analytical errors, reporting errors, or specific events that took place around the time of sampling, resulting in high concentrations. Therefore, both the frequency of detection, as well as the representativeness of a certain class or compound in monitoring campaigns, were also correlated with the RQ values, as presented in Figure 8 and Figure 11, highlighting six distinct groups of compounds that ought to be considered as hotspots, namely the **overlapping compounds** between the frequently detected and high- or medium-risk pharmaceuticals (Figure 8), as well as the **overlapping compounds** between the high- or medium-risk pharmaceuticals with the under-analyzed and inconclusive groups (Figure 11). Finally, while most metabolites included in monitoring literature were under-represented compared to parent compounds, analysis of selected metabolites with sufficient measurements allowed the calculation of RQ values, which were shown to exceed the RQ value of the parent compound. To that end, an additional assessment of possible metabolites and transformation products of the most frequently detected pharmaceuticals ought to be performed (using one of the several available in silico prediction tools, e.g., [138,139,140,141,142]), which can be correlated with the reported PNEC values for individual metabolites, in order to create a priority list for monitoring.

Several methodological limitations should be acknowledged when interpreting consumption–detection relationships. First, the assumption of proportionality between pharmaceutical usage and environmental detection does not account for differences in metabolism, excretion rates, WWTP removal efficiencies, or environmental persistence, all of which vary widely across therapeutic classes and individual APIs. Second, the available monitoring data are heterogeneous across studies, with considerable variation in sampling locations, analytical methods, seasonal coverage, and detection limits. This restricts direct calculation of numerical correlation coefficients and necessitates reliance on qualitative alignment categories. Third, many metabolites and transformation products lack reported PNECs or were monitored only once or twice, limiting the robustness of their risk characterization. Finally, consumption data from the OECD are aggregated in therapeutic classes, thus not allowing investigation of the consumption patterns of individual compounds of interest, and exclude non-reimbursed and OTC medications, which likely leads to underestimation of real consumption for certain high-frequency pollutants, such as NSAIDs and analgesics. These limitations should be considered when interpreting consumption, environmental detection, and ecotoxicity relationships, and when designing future monitoring programs.

## 5. Conclusions

This review reveals clear gaps between pharmaceutical consumption, environmental occurrence, and ecological risk in Greece, highlighting the need for a targeted, risk-based monitoring strategy. By correlating consumption data for different pharmaceutical classes, detection frequency, measured environmental concentrations of different pharmaceuticals in wastewater and the environment, and ecotoxicity data (i.e., calculated MEC/PNEC risk results for different identified pharmaceuticals), 17 priority substances were identified in this work, which require routine monitoring in wastewater and the environment. These include the NSAIDs diclofenac, ibuprofen, and naproxen; the antibiotics ciprofloxacin, moxifloxacin, azithromycin, clarithromycin, and metronidazole; the biocide triclosan; the contrast agents diatrizoic acid and iopamidol; the hormones 17α-ethinyl estradiol and etonogestrel; and the pharmaceuticals valproic acid, gemfibrozil, and sertraline and its metabolite O-desmethylvenlafaxine.

To make monitoring more efficient, it is recommended to include these priority compounds in at least quarterly monitoring of WWTP influent, effluent, and downstream surface waters. NSAIDs, macrolide and fluoroquinolone antibiotics, and synthetic estrogens should be monitored monthly because they consistently show high RQ values. In addition, ATC classes A (alimentary tract) and B (blood and blood-forming organs) need broader analytical coverage, as they are widely consumed but remain largely absent from current environmental monitoring data. Finally, some metabolites demonstrate higher ecotoxicological relevance than their parent APIs yet are rarely monitored.

Future research should prioritize (i) acquiring specific consumption data per API, instead of aggregated data per therapeutic group, particularly for classes with high consumption and ecotoxicity potential; (ii) systematic inclusion of major human metabolites in monitoring; (iii) improved national data on improper disposal of pharmaceuticals; and (iv) seasonal and targeted monitoring to capture consumption or detection spikes (e.g., tourism-driven consumption, accidental release of pharmaceuticals). Overall, these findings support better alignment of national monitoring programs with upcoming EU requirements (WFD Watch List; Urban Wastewater Treatment Directive 2024/3019) and can help Greece allocate monitoring resources more efficiently in future wastewater surveillance efforts.

## Figures and Tables

**Table 1:** Summary of priority pharmaceutical compounds, grouped by therapeutic pharmaceutical classes, key environmental risk drivers, and recommended monitoring frequencies. Exposure and effect categories were defined using a percentile-based screening approach, with high exposure defined as MEC > 95th percentile, moderate exposure as MEC between the 50th and 95th percentiles, low effect thresholds as PNEC < 25th percentile, and moderate effect thresholds between the 25th and 50th percentiles.

Pharmaceutical Class	Priority Compounds	Key Risk Drivers	Recommended Monitoring Frequency
Class M01A	Diclofenac	Moderate MEC Low PNEC	Monthly
	Ibuprofen	High MEC Low PNEC	Monthly
	Naproxen	High MEC	Monthly
Class J01	Ciprofloxacin	Moderate MEC Low PNEC	Monthly
	Moxifloxacin	Moderate MEC Moderate PNEC	Monthly
	Azithromycin	Moderate MEC Low PNEC	Monthly
	Clarithromycin	Moderate MEC Moderate PNEC	Monthly
	Metronidazole	Moderate PNEC	Quarterly
NOT IN LIST (Biocide)	Triclosan	Moderate MEC	Quarterly
NOT IN LIST (Contrast agents)	Diatrizoic acid	Moderate MEC Low PNEC	Quarterly
	Iopamidol	Low PNEC	Quarterly
Class G03	17α-ethinyl estradiol	Low PNEC	Monthly
	Etonogestrel	Low PNEC	Quarterly
Class N	Valproic acid	High MEC	Quarterly
Class N06A	Sertraline	Moderate PNEC	Quarterly
	O-desmethylvenlafaxine	Moderate MEC	Quarterly
Class C10	Gemfibrozil	Moderate MEC	Quarterly

## Data Availability

The raw data supporting the conclusions of this article will be made available by the authors upon request.

## Supplementary Materials

The following supporting information can be downloaded at: https://www.mdpi.com/article/10.3390/toxics14010045/s1, Table S1: Overview of identified APIs, metabolites, and transformation products, including their therapeutic class, monitoring and detection frequency, and maximum concentrations reported in wastewater and natural waters in Greece; Table S2: Overview of the ecotoxicity data for all identified APIs, metabolites, and transformation products, including lowest PNEC values, maximum concentrations measured in wastewater and natural waters in Greece, calculated RQs with associated risk categories, detection ratios, and corresponding monitoring classifications for each compound.

## Abbreviations

The following abbreviations are used in this manuscript:

API	Active Pharmaceutical Ingredient
ATC	Anatomical Therapeutic Chemical
CRED	Criteria for Reporting and Evaluating Ecotoxicity Data
EOPY	National Organization for the Provision of Health Services
IOBE	Economic and Industrial Research Foundation
LOD	Limit of Detection
MEC	Measured Environmental Concentration
NSAIDs	Nonsteroidal Anti-Inflammatory Drugs
OECD	Organization for Economic Co-operation and Development
OTC	Over the Counter
PNEC	Predicted No-Effect Concentration
R&D	Research and Development
RQ	Risk Quotient
SfEE	Hellenic Association of Pharmaceutical Companies
SuSDat	Substance Database
WWTPs	Wastewater Treatment Plant

## References

[1] Statista Website Pharmaceutical Market Worldwide Revenue 2001–2021. https://www.statista.com/statistics/263102/pharmaceutical-market-worldwide-revenue-since-2001/.

[2] Statista Website Worldwide Pharmaceutical R&D Spending 2014–2028. https://www.statista.com/statistics/309466/global-r-and-d-expenditure-for-pharmaceuticals/.

[3] Statista Website CDER Drug Approvals U.S. 2008–2022. https://www.statista.com/statistics/817534/annual-novel-drug-approvals-by-cder/.

[4] IQVIA Institute for Human Data Science (2025). Global Trends in R&D 2025: Progress in Recapturing Momentum in Biopharma Innovation. https://www.iqvia.com/insights/the-iqvia-institute/reports-and-publications/reports/global-trends-in-r-and-d-2025.

[5] United Nations Website World Population Projected to Reach 9.8 Billion in 2050, and 11.2 Billion in 2100. https://www.un.org/en/desa/world-population-projected-reach-98-billion-2050-and-112-billion-2100.

[6] Wernet G., Conradt S., Isenring H.P., Jiménez-González C., Hungerbühler K. (2010). Life cycle assessment of fine chemical production: A case study of pharmaceutical synthesis. Int. J. Life Cycle Assess.

[7] De Soete W., Jiménez-González C., Dahlin P., Dewulf J. (2017). Challenges and recommendations for environmental sustainability assessments of pharmaceutical products in the healthcare sector. Green Chem..

[8] Jeswani H.K., Azapagic A. (2020). Environmental impacts of healthcare and pharmaceutical products: Influence of product design and consumer behaviour. J. Clean. Prod..

[9] Bartolo N.S., Azzopardi L.M., Serracino-Inglott A. (2021). Pharmaceuticals and the environment. Early Hum. Dev..

[10] Galant O., Cerfeda G., McCalmont A.S., James S.L., Porcheddu A., Delogu F., Crawford D.E., Colacino E., Spatari S. (2022). Mechanochemistry can reduce life cycle environmental impacts of manufacturing active pharmaceutical ingredients. ACS Sustain. Chem. Eng..

[11] Jimenez-Gonzalez C., Curzons A.D., Constable D.J., Cunningham V.L. (2005). Expanding GSK’s solvent selection guide—Application of life cycle assessment to enhance solvent selections. Clean Technol. Environ. Policy.

[12] Henderson R.K., Jiménez-González C., Preston C., Constable D.J., Woodley J.M. (2008). Peer review original research: EHS & LCA assessment for 7-ACA synthesis A case study for comparing biocatalytic & chemical synthesis. Ind. Biotechnol..

[13] Jiménez-González C., Overcash M.R. (2000). Energy optimization during early drug development and the relationship with environmental burdens. J. Chem. Technol. Biotechnol..

[14] Lee C.K., Khoo H.H., Tan R.B. (2016). Life cyle assessment based environmental performance comparison of batch and continuous processing: A case of 4-D-erythronolactone synthesis. Org. Process Res. Dev..

[15] Kümmerer K. (2001). Drugs in the environment: Emission of drugs, diagnostic aids and disinfectants into wastewater by hospitals in relation to other sources—A review. Chemosphere.

[16] Santos L.H., Araújo A.N., Fachini A., Pena A., Delerue-Matos C., Montenegro M.C.B.S.M. (2010). Ecotoxicological aspects related to the presence of pharmaceuticals in the aquatic environment. J. Hazard. Mater..

[17] Li W.C. (2014). Occurrence, sources, and fate of pharmaceuticals in aquatic environment and soil. Environ. Pollut..

[18] Boxall A.B., Rudd M.A., Brooks B.W., Caldwell D.J., Choi K., Hickmann S., Innes E., Ostapyk K., Staveley J.P., Verslycke T. (2012). Pharmaceuticals and personal care products in the environment: What are the big questions?. Environ. Health Perspect..

[19] Bu Q., Shi X., Yu G., Huang J., Wang B. (2016). Assessing the persistence of pharmaceuticals in the aquatic environment: Challenges and needs. Emerg. Contam..

[20] Bu Q., Shi X., Yu G., Huang J., Wang B., Wang J. (2016). Pay attention to non-wastewater emission pathways of pharmaceuticals into environments. Chemosphere.

[21] Mudgal S., De Toni A., Lockwood S., Salès K., Backhaus T., Sorensen B.H. (2013). Study on the Environmental Risks of Medicinal Products. Doctoral Dissertation.

[22] Emara Y., Lehmann A., Siegert M.W., Finkbeiner M. (2019). Modeling pharmaceutical emissions and their toxicity-related effects in life cycle assessment (LCA): A review. Integr. Environ. Assess Manag..

[23] Kanakaraju D., Glass B.D., Oelgemöller M. (2018). Advanced oxidation process-mediated removal of pharmaceuticals from water: A review. J. Environ. Manag..

[24] Kadmi Y., Favier L., Wolbert D. (2015). N-nitrosamines, emerging disinfection by-products of health concern: An overview of occurrence, mechanisms of formation, control and analysis in water. Water Sci. Technol. Water Supply.

[25] Osorio V., Sanchís J., Abad J.L., Ginebreda A., Farré M., Pérez S., Barceló D. (2016). Investigating the formation and toxicity of nitrogen transformation products of diclofenac and sulfamethoxazole in wastewater treatment plants. J. Hazard. Mater..

[26] Lonappan L., Brar S.K., Das R.K., Verma M., Surampalli R.Y. (2016). Diclofenac and its transformation products: Environmental occurrence and toxicity—A review. Environ. Int..

[27] Han E.J., Lee D.S. (2017). Significance of metabolites in the environmental risk assessment of pharmaceuticals consumed by human. Sci. Total Environ..

[28] Grabarczyk Ł., Mulkiewicz E., Stolte S., Puckowski A., Pazda M., Stepnowski P., Białk-Bielińska A. (2020). Ecotoxicity screening evaluation of selected pharmaceuticals and their transformation products towards various organisms. Environ. Sci. Pollut. Res..

[29] Fatta-Kassinos D., Vasquez M.I., Kümmerer K. (2011). Transformation products of pharmaceuticals in surface waters and wastewater formed during photolysis and advanced oxidation processes–degradation, elucidation of byproducts and assessment of their biological potency. Chemosphere.

[30] Brown A.K., Wong C.S. (2018). Distribution and fate of pharmaceuticals and their metabolite conjugates in a municipal wastewater treatment plant. Water Res..

[31] Al Aukidy M., Verlicchi P., Voulvoulis N. (2014). A framework for the assessment of the environmental risk posed by pharmaceuticals originating from hospital effluents. Sci. Total Environ..

[32] Bailly E., Levi Y., Karolak S. (2013). Calibration and field evaluation of polar organic chemical integrative sampler (POCIS) for monitoring pharmaceuticals in hospital wastewater. Environ. Pollut..

[33] Daouk S., Chèvre N., Vernaz N., Widmer C., Daali Y., Fleury-Souverain S. (2016). Dynamics of active pharmaceutical ingredients loads in a Swiss university hospital wastewaters and prediction of the related environmental risk for the aquatic ecosystems. Sci. Total Environ..

[34] Escher B.I., Baumgartner R., Koller M., Treyer K., Lienert J., McArdell C.S. (2011). Environmental toxicology and risk assessment of pharmaceuticals from hospital wastewater. Water Res..

[35] Frédéric O., Yves P. (2014). Pharmaceuticals in hospital wastewater: Their ecotoxicity and contribution to the environmental hazard of the effluent. Chemosphere.

[36] Helwig K., Hunter C., MacLachlan J., McNaughtan M., Roberts J., Cornelissen A., Dagot C., Evenblij H., Klepiszewski K., Lyko S. (2013). Micropollutant point sources in the built environment: Identification and monitoring of priority pharmaceutical substances in hospital effluents. J. Environ. Anal. Toxicol..

[37] Kümmerer K., Henninger A. (2013). Promoting resistance by the emission of antibiotics from hospitals and households into effluent. Clin. Microbiol. Infect..

[38] Pérez-Alvarez I., Islas-Flores H., Gómez-Oliván L.M., Barceló D., De Alda M.L., Solsona S.P., Sánchez-Aceves L., SanJuan-Reyes N., Galar-Martínez M. (2018). Determination of metals and pharmaceutical compounds released in hospital wastewater from Toluca, Mexico, and evaluation of their toxic impact. Environ. Pollut..

[39] Ortner P., McCullagh M. (2010). Hospice nurses and drug disposal: The convergence between nursing practice and the environment. J. Hosp. Palliat. Nurs..

[40] Blair B.D. (2016). Potential Upstream Strategies for the Mitigation of Pharmaceuticals in the Aquatic Environment: A Brief Review. Curr. Environ. Health Rep..

[41] Lyko S., Nafo I., Evenblij H., Benetto E., Cornelissen A., Igos E., Klepiszewski K., Venditti S., Kovalova L., McArdell C. (2012). Pharmaceutical Input and Elimination from local sources. Final Report of the European Cooperation Project PILLS.

[42] Paulus G.K., Hornstra L.M., Alygizakis N., Slobodnik J., Thomaidis N., Medema G. (2019). The impact of on-site hospital wastewater treatment on the downstream communal wastewater system in terms of antibiotics and antibiotic resistance genes. Int. J. Hyg. Environ. Health.

[43] Xiang J., Wu M., Lei J., Fu C., Gu J., Xu G. (2018). The fate and risk assessment of psychiatric pharmaceuticals from psychiatric hospital effluent. Ecotoxicol. Environ. Saf..

[44] Chonova T., Lecomte V., Bertrand-Krajewski J.L., Bouchez A., Labanowski J., Dagot C., Lévi Y., Perrodin Y., Wiest L., Gonzalez-Ospina A. (2018). The SIPIBEL project: Treatment of hospital and urban wastewater in a conventional urban wastewater treatment plant. Environ. Sci. Pollut. Res..

[45] de Wilt H.A. (2018). Pharmaceutical Removal: Synergy Between Biological and Chemical Processes for Wastewater Treatment. Doctoral Thesis.

[46] Petrie B. (2021). A review of combined sewer overflows as a source of wastewater-derived emerging contaminants in the environment and their management. Environ. Sci. Pollut. Res..

[47] Gadupudi C.K., Rice L., Xiao L., Kantamaneni K. (2021). Endocrine disrupting compounds removal methods from wastewater in the United Kingdom: A review. Science.

[48] Caldwell D.J., Hester R.E., Harrison R.M. (2015). Sources of Pharmaceutical Residues in the Environment and their Control. Issues in Environmental Science and Technology—Pharmaceuticals in the Environment.

[49] Szymańska U., Wiergowski M., Sołtyszewski I., Kuzemko J., Wiergowska G., Woźniak M.K. (2019). Presence of antibiotics in the aquatic environment in Europe and their analytical monitoring: Recent trends and perspectives. Microchem. J..

[50] Stankiewicz A., Giebułtowicz J., Stankiewicz U., Wroczyński P., Nałęcz-Jawecki G. (2015). Determination of selected cardiovascular active compounds in environmental aquatic samples–methods and results, a review of global publications from the last 10 years. Chemosphere.

[51] Küster A., Adler N. (2014). Pharmaceuticals in the environment: Scientific evidence of risks and its regulation. Philos. Trans. R. Soc. B Biol. Sci..

[52] Kümmerer K. (2009). Antibiotics in the aquatic environment—A review—Part I. Chemosphere.

[53] Kümmerer K. (2009). Antibiotics in the aquatic environment—A review—Part II. Chemosphere.

[54] Janecko N., Pokludova L., Blahova J., Svobodova Z., Literak I. (2016). Implications of fluoroquinolone contamination for the aquatic environment—A review. Environ. Toxicol. Chem..

[55] Hernando M.D., Mezcua M., Fernández-Alba A.R., Barceló D. (2006). Environmental risk assessment of pharmaceutical residues in wastewater effluents, surface waters and sediments. Talanta.

[56] Godoy A.A., Kummrow F., Pamplin P.A.Z. (2015). Occurrence, ecotoxicological effects and risk assessment of antihypertensive pharmaceutical residues in the aquatic environment—A review. Chemosphere.

[57] The European Union (2024). Directive (EU) 2024/3019 of the European Parliament and of the Council of 27 November 2024 Concerning Urban Wastewater Treatment (Recast).

[58] Zenker A., Cicero M.R., Prestinaci F., Bottoni P., Carere M. (2014). Bioaccumulation and biomagnification potential of pharmaceuticals with a focus to the aquatic environment. J. Environ. Manag..

[59] Bottoni P., Caroli S. (2018). Presence of residues and metabolites of pharmaceuticals in environmental compartments, food commodities and workplaces: A review spanning the three-year period 2014–2016. Microchem. J..

[60] Anand U., Adelodun B., Cabreros C., Kumar P., Suresh S., Dey A., Ballesteros F., Bontempi E. (2022). Occurrence, transformation, bioaccumulation, risk and analysis of pharmaceutical and personal care products from wastewater: A review. Environ. Chem. Lett..

[61] Malchi T., Maor Y., Tadmor G., Shenker M., Chefetz B. (2014). Irrigation of root vegetables with treated wastewater: Evaluating uptake of pharmaceuticals and the associated human health risks. Environ. Sci. Technol..

[62] Golovko O., Kaczmarek M., Asp H., Bergstrand K.J., Ahrens L., Hultberg M. (2022). Uptake of perfluoroalkyl substances, pharmaceuticals, and parabens by oyster mushrooms (*Pleurotus ostreatus*) and exposure risk in human consumption. Chemosphere.

[63] Cortes G., Rodriguez P., Marinov E., Sanseverino D., Lettieri T. (2025). Selection of Substances for the 5th Watch List Under the Water Framework Directive.

[64] Gonzalez Pena O.I., López Zavala M.Á., Cabral Ruelas H. (2021). Pharmaceuticals market, consumption trends and disease incidence are not driving the pharmaceutical research on water and wastewater. Int. J. Environ. Res. Public Health.

[65] OECD (2019). Health at a Glance 2019: OECD Indicators.

[66] Kadam A., Patil S., Patil S., Tumkur A. (2016). Pharmaceutical waste management an overview. IJOPP.

[67] OECD Website Pharmaceutical Spending. https://www.oecd.org/en/data/indicators/pharmaceutical-spending.html.

[68] European Centre for Disease Prevention and Control, Stockholm (2014). Summary of the Latest Data on Antibiotic Consumption in the European Union. https://www.ecdc.europa.eu/sites/default/files/documents/antibiotics-consumption-EU-data-2014.pdf.

[69] Mitkidis K., Obolevich V., Chrysochou P., Mitkidis P. (2021). Harmonisation of pharmaceutical take-back systems in the EU. Eur. J. Health Law.

[70] European Environment Agency Website (2000). Proportion of Terrestrial Land Covered by Natura. https://www.eea.europa.eu/en/analysis/maps-and-charts/proportion-of-terrestrial-land-covered.

[71] Mitchell D., Vulcano A., Dias M. (2022). Assessment of the Protection of Important Bird and Biodiversity Areas for Seabirds by Special Protection Areas of the Natura 2000 Network.

[72] Eurostat Website Net Occupancy Rates of Bed Places in Hotels and Similar Accommodation Establishments in the Peak Month, Summer Season 2018 (%). https://ec.europa.eu/eurostat/statistics-explained/index.php?title=File:Net_occupancy_rates_of_bed_places_in_hotels_and_similar_accommodation_establishments_in_the_peak_month,_summer_season_2018_(%25).png.

[73] Hellenic Association of Pharmaceutical Companies (SFEE) Website. https://www.sfee.gr/wp-content/uploads/2022/06/F-F-2021.pdf.

[74] Foundation for Economic & Industrial Research (IOBE) Website. https://iobe.gr/wp-content/uploads/2022/05/RES_05_A_24062021_REP_GR.pdf.

[75] Alnahas F., Yeboah P., Fliedel L., Abdin A.Y., Alhareth K. (2020). Expired medication: Societal, regulatory and ethical aspects of a wasted opportunity. Int. J. Environ. Res. Public Health.

[76] Health Care Without Harm Europe Website. https://europe.noharm.org/sites/default/files/documents-files/4646/2013-12%20Unused%20pharmaceuticals.pdf.

[77] Hellenic Government Website. Government Gazette 287Β_2007. https://www.elinyae.gr/ethniki-nomothesia/ya-86682007-fek-287b-232007.

[78] OECD Website Pharmaceutical Market Dataset. https://stats.oecd.org/viewhtml.aspx?datasetcode=HEALTH_PHMC&lang=en.

[79] Alygizakis N.A., Gago-Ferrero P., Borova V.L., Pavlidou A., Hatzianestis I., Thomaidis N.S. (2016). Occurrence and spatial distribution of 158 pharmaceuticals, drugs of abuse and related metabolites in offshore seawater. Sci. Total Environ..

[80] Andreozzi R., Raffaele M., Nicklas P. (2003). Pharmaceuticals in STP effluents and their solar photodegradation in aquatic environment. Chemosphere.

[81] Antoniou C.V., Koukouraki E.E., Diamadopoulos E. (2009). Analysis of selected pharmaceutical compounds and endocrine disruptors in municipal wastewater using solid-phase microextraction and gas chromatography. Water Environ. Res.

[82] Arditsoglou A., Voutsa D. (2008). Passive sampling of selected endocrine disrupting compounds using polar organic chemical integrative samplers. Environ. Pollut..

[83] Arditsoglou A., Voutsa D. (2008). Determination of phenolic and steroid endocrine disrupting compounds in environmental matrices. ESPR.

[84] Arditsoglou A., Voutsa D. (2010). Partitioning of endocrine disrupting compounds in inland waters and wastewaters discharged into the coastal area of Thessaloniki, Northern Greece. ESPR.

[85] Arditsoglou A., Voutsa D. (2012). Occurrence and partitioning of endocrine-disrupting compounds in the marine environment of Thermaikos Gulf, Northern Aegean Sea, Greece. Mar. Pollut. Bull..

[86] Borova V.L., Maragou N.C., Gago-Ferrero P., Pistos C., Thomaidis N.S. (2014). Highly sensitive determination of 68 psychoactive pharmaceuticals, illicit drugs, and related human metabolites in wastewater by liquid chromatography–tandem mass spectrometry. Anal. Bioanal. Chem..

[87] Botitsi E., Frosyni C., Tsipi D. (2007). Determination of pharmaceuticals from different therapeutic classes in wastewaters by liquid chromatography–electrospray ionization–tandem mass spectrometry. Anal. Bioanal. Chem..

[88] Chanioti A., Botitsi H., Antoniou S., Dasenakis E., Tsipi D. Development of a Multi-class Analytical Method by SPE and LC-MS/MS for the Determination of Pharmaceuticals in Wastewater Samples. Proceedings of the 15th International Conference on Environmental Science and Technology.

[89] Christophoridis C., Veloutsou S., Mitsika E., Zacharis C.K., Christia C., Raikos N., Fytianos K. (2021). Determination of illicit drugs and psychoactive pharmaceuticals in wastewater from the area of Thessaloniki (Greece) using LC–MS/MS: Estimation of drug consumption. Environ. Monit. Assess.

[90] Dasenaki M.E., Thomaidis N.S. (2015). Multianalyte method for the determination of pharmaceuticals in wastewater samples using solid-phase extraction and liquid chromatography–tandem mass spectrometry. Anal. Bioanal. Chem..

[91] Gago-Ferrero P., Borova V., Dasenaki M.E., Thomaidis N.S. (2015). Simultaneous determination of 148 pharmaceuticals and illicit drugs in sewage sludge based on ultrasound-assisted extraction and liquid chromatography–tandem mass spectrometry. Anal. Bioanal. Chem..

[92] Galani A., Alygizakis N., Aalizadeh R., Kastritis E., Dimopoulos M.A., Thomaidis N.S. (2021). Patterns of pharmaceuticals use during the first wave of COVID-19 pandemic in Athens, Greece as revealed by wastewater-based epidemiology. Sci. Total Environ..

[93] Gatidou G., Thomaidis N.S., Stasinakis A.S., Lekkas T.D. (2007). Simultaneous determination of the endocrine disrupting compounds nonylphenol, nonylphenol ethoxylates, triclosan and bisphenol A in wastewater and sewage sludge by gas chromatography–mass spectrometry. J. Chromatogr. A.

[94] Ibáñez M., Borova V., Boix C., Aalizadeh R., Bade R., Thomaidis N.S., Hernandez F. (2017). UHPLC-QTOF MS screening of pharmaceuticals and their metabolites in treated wastewater samples from Athens. J. Hazard. Mater..

[95] Kalaboka M., Chrimatopoulos C., Jimenez-Holgado C., Boti V., Sakkas V., Albanis T. (2020). Exploring the Efficiency of UHPLC-Orbitrap MS for the Determination of 20 Pharmaceuticals and Acesulfame K in Hospital and Urban Wastewaters with the Aid of FPSE. Separations.

[96] Konstas P.S., Kosma C., Konstantinou I., Albanis T. (2019). Photocatalytic treatment of pharmaceuticals in real hospital wastewaters for effluent quality amelioration. Water.

[97] Kosma C.I., Lambropoulou D.A., Albanis T.A. (2010). Occurrence and removal of PPCPs in municipal and hospital wastewaters in Greece. J. Hazard. Mater..

[98] Kosma C.I., Lambropoulou D.A., Albanis T.A. (2014). Investigation of PPCPs in wastewater treatment plants in Greece: Occurrence, removal and environmental risk assessment. Sci. Total Environ..

[99] Kosma C.I., Lambropoulou D.A., Albanis T.A. (2015). Comprehensive study of the antidiabetic drug metformin and its transformation product guanylurea in Greek wastewaters. Water Res..

[100] Kosma C.I., Nannou C.I., Boti V.I., Albanis T.A. (2019). Psychiatrics and selected metabolites in hospital and urban wastewaters: Occurrence, removal, mass loading, seasonal influence and risk assessment. Sci. Total Environ..

[101] Kotti M., Papafilippaki A., Stavroulakis G. (2021). Simultaneous determination of selected pharmaceuticals and plasticisers in urban stormwater in Chania (Greece). Int. J. Environ. Anal. Chem..

[102] Koumaki E., Mamais D., Noutsopoulos C. (2017). Environmental fate of non-steroidal anti-inflammatory drugs in river water/sediment systems. J. Hazard. Mater..

[103] Koumaki E., Mamais D., Noutsopoulos C. (2018). Assessment of the environmental fate of endocrine disrupting chemicals in rivers. Sci. Total Environ..

[104] Koutsouba V., Heberer T., Fuhrmann B., Schmidt-Baumler K., Tsipi D., Hiskia A. (2003). Determination of polar pharmaceuticals in sewage water of Greece by gas chromatography–mass spectrometry. Chemosphere.

[105] Loos R., Gawlik B.M., Locoro G., Rimaviciute E., Contini S., Bidoglio G. (2008). EU-wide monitoring survey of polar persistent pollutants in European river waters. Environ. Pollut..

[106] Loos R., Carvalho R., António D.C., Comero S., Locoro G., Tavazzi S., Paracchini B., Ghiani M., Lettieri T., Blaha L. (2013). EU-wide monitoring survey on emerging polar organic contaminants in wastewater treatment plant effluents. Water Res..

[107] Mandaric L., Kalogianni E., Skoulikidis N., Petrovic M., Sabater S. (2019). Contamination patterns and attenuation of pharmaceuticals in a temporary Mediterranean river. Sci. Total Environ..

[108] Miserli K., Nastopoulou A., Konstantinou I. (2022). Removal of organic pollutants (pharmaceuticals and pesticides) from sewage sludge by hydrothermal carbonization using response surface methodology (RSM). J. Chem. Technol. Biotechnol..

[109] Nannou C.I., Kosma C.I., Albanis T.A. (2015). Occurrence of pharmaceuticals in surface waters: Analytical method development and environmental risk assessment. Int. J. Environ. Anal. Chem..

[110] Nannou C.I., Boti V., Albanis T. Accurate Mass Screening of Pharmaceuticals in Water and Sediment by Uhplc-Orbitrap Mass Spectrometry. Proceedings of the 15th International Conference on Environmental Science and Technology.

[111] Nannou C.I., Boti V.I., Albanis T.A. (2019). A modified QuEChERS approach for the analysis of pharmaceuticals in sediments by LC-Orbitrap HRMS. Anal. Bioanal. Chem..

[112] Nannou C., Kaprara E., Psaltou S., Salapasidou M., Palasantza P.A., Diamantopoulos P., Lambropoulou D.A., Mitrakas M., Zouboulis A. (2022). Monitoring of a broad set of pharmaceuticals in wastewaters by high-resolution mass spectrometry and evaluation of heterogenous catalytic ozonation for their removal in a pre-industrial level unit. Analytica.

[113] Nödler K., Voutsa D., Licha T. (2014). Polar organic micropollutants in the coastal environment of different marine systems. Mar. Pollut. Bull..

[114] Nödler K., Tsakiri M., Aloupi M., Gatidou G., Stasinakis A.S., Licha T. (2016). Evaluation of polar organic micropollutants as indicators for wastewater-related coastal water quality impairment. Environ. Pollut..

[115] Noutsopoulos C., Koumaki E., Sarantopoulos V., Mamais D. (2019). Analytical and mathematical assessment of emerging pollutants fate in a river system. J. Hazard. Mater..

[116] Ofrydopoulou A., Nannou C., Evgenidou E., Christodoulou A., Lambropoulou D. (2022). Assessment of a wide array of organic micropollutants of emerging concern in wastewater treatment plants in Greece: Occurrence, removals, mass loading and potential risks. Sci. Total Environ..

[117] Papageorgiou M., Kosma C., Lambropoulou D. (2016). Seasonal occurrence, removal, mass loading and environmental risk assessment of 55 pharmaceuticals and personal care products in a municipal wastewater treatment plant in Central Greece. Sci. Total Environ..

[118] Papageorgiou M., Zioris I., Danis T., Bikiaris D., Lambropoulou D. (2019). Comprehensive investigation of a wide range of pharmaceuticals and personal care products in urban and hospital wastewaters in Greece. Sci. Total Environ..

[119] Pothitou P., Voutsa D. (2008). Endocrine disrupting compounds in municipal and industrial wastewater treatment plants in Northern Greece. Chemosphere.

[120] Samaras V.G., Thomaidis N.S., Stasinakis A.S., Gatidou G., Lekkas T.D. (2010). Determination of selected non-steroidal anti-inflammatory drugs in wastewater by gas chromatography-mass spectrometry. Int. J. Environ. Anal. Chem..

[121] Samaras V.G., Thomaidis N.S., Stasinakis A.S., Lekkas T.D. (2011). An analytical method for the simultaneous trace determination of acidic pharmaceuticals and phenolic endocrine disrupting chemicals in wastewater and sewage sludge by gas chromatography-mass spectrometry. Anal. Bioanal. Chem..

[122] Samaras V.G., Stasinakis A.S., Mamais D., Thomaidis N.S., Lekkas T.D. (2013). Fate of selected pharmaceuticals and synthetic endocrine disrupting compounds during wastewater treatment and sludge anaerobic digestion. J. Hazard. Mater..

[123] Stamatis N.K., Konstantinou I.K. (2013). Occurrence and removal of emerging pharmaceutical, personal care compounds and caffeine tracer in municipal sewage treatment plant in Western Greece. J. Environ. Sci. Health B.

[124] Stamatis N., Hela D., Konstantinou I. (2010). Occurrence and removal of fungicides in municipal sewage treatment plant. J. Hazard. Mater..

[125] Stamatis N., Triantafyllidis V., Hela D., Konstantinou I. (2013). Occurrence and distribution of selected pharmaceutical compounds on sewage-impacted section of River Acheloos, Western Greece. Int. J. Environ. Anal. Chem..

[126] Stasinakis A.S., Gatidou G., Mamais D., Thomaidis N.S., Lekkas T.D. (2008). Occurrence and fate of endocrine disrupters in Greek sewage treatment plants. Water Res..

[127] Stasinakis A.S., Mermigka S., Samaras V.G., Farmaki E., Thomaidis N.S. (2012). Occurrence of endocrine disrupters and selected pharmaceuticals in Aisonas River (Greece) and environmental risk assessment using hazard indexes. Environ. Sci. Pollut. Res..

[128] Stasinakis A.S., Thomaidis N.S., Arvaniti O.S., Asimakopoulos A.G., Samaras V.G., Ajibola A., Mamais D., Lekkas T.D. (2013). Contribution of primary and secondary treatment on the removal of benzothiazoles, benzotriazoles, endocrine disruptors, pharmaceuticals and perfluorinated compounds in a sewage treatment plant. Sci. Total Environ..

[129] Terzopoulou E., Voutsa D., Kaklamanos G. (2015). A multi-residue method for determination of 70 organic micropollutants in surface waters by solid-phase extraction followed by gas chromatography coupled to tandem mass spectrometry. Environ. Sci. Pollut. Res..

[130] Thomaidi V.S., Stasinakis A.S., Borova V.L., Thomaidis N.S. (2015). Is there a risk for the aquatic environment due to the existence of emerging organic contaminants in treated domestic wastewater? Greece as a case-study. J. Hazard. Mater..

[131] Thomaidi V.S., Stasinakis A.S., Borova V.L., Thomaidis N.S. (2016). Assessing the risk associated with the presence of emerging organic contaminants in sludge-amended soil: A country-level analysis. Sci. Total Environ..

[132] Thomaidis N.S., Gago-Ferrero P., Ort C., Maragou N.C., Alygizakis N.A., Borova V.L., Dasenaki M.E. (2016). Reflection of socioeconomic changes in wastewater: Licit and illicit drug use patterns. Environ. Sci. Technol..

[133] Wang C., Ye D., Li X., Jia Y., Zhao L., Liu S., Xu J., Du J., Tian L., Li J. (2021). Occurrence of pharmaceuticals and personal care products in bottled water and assessment of the associated risks. Environ. Int..

[134] NORMAN Database System Website. https://www.norman-network.com/nds/.

[135] Dulio V., Koschorreck J., Van Bavel B., Van Den Brink P., Hollender J., Munthe J., Schlabach M., Aalizadeh R., Agerstrand M., Ahrens L. (2020). The NORMAN Association and the European Partnership for Chemicals Risk Assessment (PARC): Let’s Cooperate!. Environ. Sci. Eur..

[136] ClinPGx Database Website. https://www.clinpgx.org.

[137] Yang Q., Gao Y., Ke J., Show P.L., Ge Y., Liu Y., Guo R., Chen J. (2021). Antibiotics: An overview on the environmental occurrence, toxicity, degradation, and removal methods. Bioengineered.

[138] Zhu K., Huang M., Wang Y., Gu Y., Li W., Liu G., Tang Y. (2024). MetaPredictor: In silico prediction of drug metabolites based on deep language models with prompt engineering. Brief Bioinform..

[139] Kazmi S.R., Jun R., Yu M.S., Jung C., Na D. (2019). In silico approaches and tools for the prediction of drug metabolism and fate: A review. Comput. Biol. Med..

[140] Litsa E.E., Das P., Kavraki L.E. (2020). Prediction of drug metabolites using neural machine translation. Chem. Sci..

[141] Smith A.M.E., Lanevskij K., Sazonovas A., Harris J. (2022). Impact of established and emerging software tools on the metabolite identification landscape. Front. Toxicol..

[142] Litsa E.E., Das P., Kavraki L.E. (2022). Machine learning models in the prediction of drug metabolism: Challenges and future perspectives. Expert Opin. Drug Metab. Toxicol..

